# The effects of a preschool-based intervention for Early Childhood Education and Care teachers in promoting healthy eating and physical activity in young children: A cluster randomised controlled trial

**DOI:** 10.1371/journal.pone.0255023

**Published:** 2021-07-23

**Authors:** Nicole Toussaint, Martinette T. Streppel, Sandra Mul, Marielle Balledux, Karen van Drongelen, Mirka Janssen, Ruben G. Fukkink, Peter J. M. Weijs

**Affiliations:** 1 Faculty of Sports and Nutrition, Center of Expertise Urban Vitality, Amsterdam University of Applied Sciences, Amsterdam, The Netherlands; 2 Netherlands Youth Institute, Utrecht, The Netherlands; 3 The Netherlands Nutrition Centre, The Hague, The Netherlands; 4 Faculty of Child Development and Education, Amsterdam University of Applied Sciences, Amsterdam, The Netherlands; 5 Faculty of Social and Behavioural Sciences, University of Amsterdam, Amsterdam, The Netherlands; 6 Department of Nutrition & Dietetics, Amsterdam University Medical Centers, VU University, Amsterdam, The Netherlands; Weill Cornell Medical College in Qatar, QATAR

## Abstract

The need for excess weight gain prevention in disadvantaged young children is widely recognised. Early Childhood Education and Care teachers are potential key actors in early interventions to prevent overweight and obesity. This study examines the effects of a preschool-based intervention for teachers in promoting healthy eating and physical activity in young children. A cluster randomised controlled trial was conducted at 41 preschools in a deprived area of Amsterdam, The Netherlands. The intervention consisted of 2 programmes that were applied in succession: A Healthy Start and PLAYgrounds for TODdlers. The study period was 9 months. Primary outcomes were assessed via questionnaires and included teachers’ knowledge, attitude, food/activity-related practices, and level of confidence in promoting healthy behaviours. Secondary outcomes in this study were teachers’ and children’s BMI (z-score), body composition, dietary intake and physical activity level. Intention-to-treat analyses were performed using linear mixed models. In total, 115 teachers and 249 children (mean age 3.0 (0.2) years) were included. A positive effect on teachers’ knowledge about the Dutch dietary guidelines was found after the programme A Healthy Start (difference = 1.38; 1-sided 95% CL = 0.29; p = 0.02). This effect was not sustained at 9 months (difference = 0.34; 1-sided 95% CL = -0.76; p = 0.31). The overall intervention had a positive effect on 3 of the 5 attitude statements regarding a healthy lifestyle (difference ranged from 0.34 to 0.55) and on the practice scale Activity-related-Modelling (difference = 0.16; 1-sided 95% CL = 0.06; p = 0.01). No intervention effects were observed on food-related practice scales and the level of confidence in promoting healthy behaviours. At this stage, no effects were seen on teachers’ and children’s BMI (z-score). This study contributes to the professional development of Early Childhood Education and Care teachers and addresses the call for interventions to prevent overweight/obesity and to minimise health inequalities in young children.

## Introduction

Excess weight gain in children remains a critical health issue worldwide [1]. The problem is more severe in deprived urban settings, where it is related to the relatively high number of families with a migration background and/or low socio-economic status [2]. Children show health inequalities already in the preschool period, and the need for early interventions to prevent overweight or obesity in deprived areas is widely recognised [3–8].

In the Netherlands, urban preschools provide an excellent opportunity to reach many young children (2.5 to 4 years old) from families with a migration background and/or low socio-economic status. In particular, parents of children at risk for language or developmental delays are advised to enrol their child in these community-based services that provide play-based education. Children generally spend up to 15 hours per week in preschool. These Early Childhood Education and Care (ECEC) settings are therefore important environments for interventions in young children with disadvantaged backgrounds [9, 10].

Various overweight and obesity prevention interventions in ECEC settings have been reviewed. Ward et al. (2017) suggest in their systematic review that multi-component and multi-level interventions with both centre-based and home-based involvement have a most favourable effect on weight-related outcomes [11]. Early Childhood Education and Care (ECEC) teachers at urban preschools are potential key actors in the interventions to prevent overweight or obesity [12, 13]. Young children learn through observing and imitating, and next to parents, ECEC teachers may set a healthy example and promote healthy behaviours. It is important to gain insight in the teachers’ knowledge, attitude and food/activity-related practices. Moreover, their level of confidence in promoting healthy behaviours is of interest. In a recent systematic review by Zhang et al. (2018), the authors report ECEC teachers’ habitual physical activity level and body weight to be associated to the weight status of young children. However, the authors emphasise that the strength of evidence from the studies reviewed is still limited [14]. Ward et al. (2015) suggest in their systematic review a positive role for ECEC teachers in promoting healthy behaviours in children. They conclude that the effect of specific practices of ECEC teachers on healthy eating and physical activity in young children remains unclear because of the lack of high-quality intervention trials. Ward et al. recommend programme developers to also intervene in disadvantaged areas and include children from families with a migration background and/or low socio-economic status in future research to address gaps in the current evidence [15].

Our study, PreSchool@HealthyWeight, concerns an intervention study with urban preschools mainly located in Amsterdam Nieuw-West, the Netherlands. This city district is characterised by a relatively high level of inhabitants with a migration background and/or low socio-economic status [16]. In 2016, 12.0% of the children aged 3 years were reported to be overweight or obese in Amsterdam Nieuw-West, while in other city districts of Amsterdam this percentage ranged from 4.7 to 8.4 [17].

PreSchool@HealthyWeight involves ECEC teacher training and focusses on the knowledge and practices of the teachers in order to set a healthy example and create a healthy environment for young children. The primary aim is to examine whether the intervention improves the teachers’ knowledge, attitude, food/activity-related practices, and level of confidence in promoting healthy eating and physical activity. As it is hypothesised that changes in ECEC teachers eventually transfer to changes in children, the secondary objective of this study is to investigate if the intervention improves the teachers’ and children’s Body Mass Index (BMI) (z-score), body composition, dietary intake and physical activity level.

## Materials and methods

### Study design and setting

**Fig 1:**
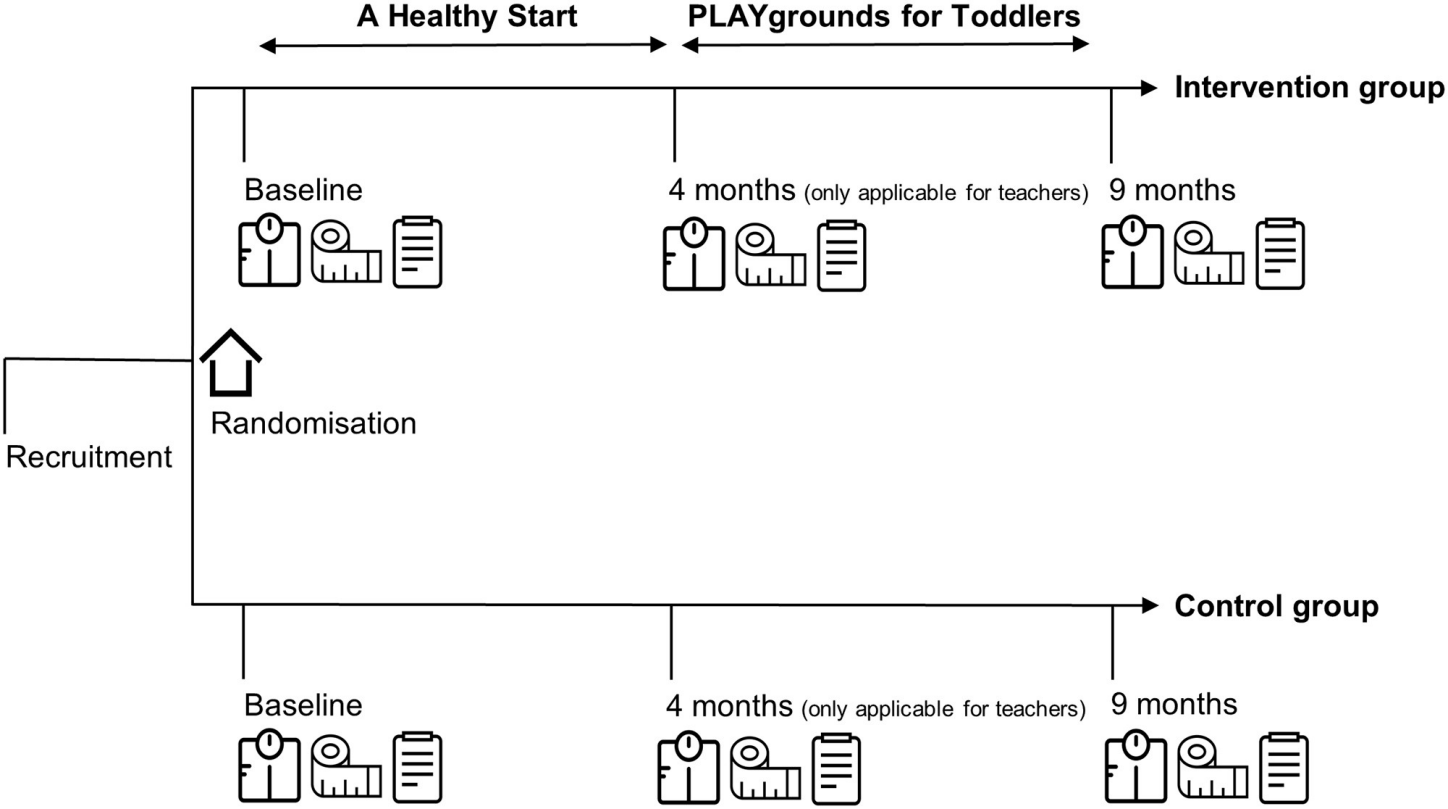
Schematic overview of the study.

Between September 2016 and January 2018, a cluster randomised controlled trial was conducted at 41 preschools. The sample comprised preschools of the largest childcare organisation in the deprived area Amsterdam Nieuw-West, the Netherlands. Included preschools (clusters) were randomly allocated to an intervention or control group. The randomisation was performed by an independent researcher with the use of computer-generated lists. The intervention for ECEC teachers consisted of modified versions of 2 existing Dutch programmes: A Healthy Start (AHS) (Dutch: Een Gezonde Start) [18] and PLAYgrounds for TODdlers (PLAYTOD) (Dutch: PLAYgrounds voor Peuters) [19]. The programmes were applied in succession. All ECEC teachers at intervention preschools were involved in the programmes (upon request of the management of the childcare organisation, as further training for their employees). However, not all ECEC teachers were involved in the study as participation was voluntarily. ECEC teachers at control preschools did not receive the intervention programmes. The childcare organisation had a healthy food policy, so also ECEC teachers from control preschools were supposed to pay attention to healthy (eating) behaviours of the children. However, it was not likely that teachers at control preschools received any professional training relating to healthy eating/physical activity behaviours during the study period, as this is not standard in (pre- or in-service) professional development of Dutch ECEC teachers and childcare policy. Participating ECEC teachers and parents were asked to fill in a questionnaire at baseline, at 4 months (only applicable for ECEC teachers) and at 9 months. At the same time, anthropometric measurements in teachers and children were carried out. Furthermore, ECEC teachers and parents were asked to self-report their own (for ECEC teachers) or their children’s (for parents) food intake and physical activities for 3 days at baseline and at 9 months. A detailed description of the study protocol was previously published [20]. Fig 1 shows a schematic overview of the study design. This study followed the CONSORT statement [21] and extension to cluster randomised trials [22]. The Medical Ethics Review Committee of the VU University Medical Center stated that the Medical Research Involving Human Subjects Act does not apply to this study (reference number: 2016.310). Trial registration: Netherlands Trial Register (NTR), NL5850 (date registered: August 26, 2016).

### Study sample and recruitment

All ECEC teachers (n = 162) at the 41 included preschools were approached via information meetings. Parents were individually approached (face-to-face) at preschools by the research staff in close cooperation with the centre. Children had to be between 2.5 to 3.5 years old to be included. All the participating ECEC teachers and parents gave written informed consent. The parents gave additional written informed consent for the participation of their child.

### Intervention

#### A Healthy Start programme (AHS)

The national AHS programme is an initiative of the Dutch Ministry of Health, Welfare and Sport and the Ministry of Social Affairs and Employment. It is developed by 7 renowned Dutch knowledge centres on different aspects of lifestyle and train-the-trainer courses are being organised. AHS focusses on the knowledge and practices of the teachers in order to be a healthy role model and create a healthy, active and safe environment for children [18]. Coaches of the participating childcare organisation composed, together with the research staff, a modified version of AHS to train their colleague ECEC teachers. A detailed description of the intervention was previously published [20]. In short, 3 face-to-face meetings of 2 hours each were organised for 8 groups of ECEC teachers. Each meeting was led by 1 of the coaches and a member of the research staff. The 3 meetings included theory and practical assignments from the basic national AHS module about a healthy childcare environment and in-depth national AHS modules about Nutrition, Physical Activity and Body weight. In the first meeting, the teachers reflected on their personal lifestyle outside preschool and the 2015 Dutch dietary guidelines were discussed. The second meeting focussed on the interaction with children regarding a healthy lifestyle, and the pivotal role of ECEC teachers in setting a healthy example. Best practices were promoted. For example, ECEC teachers learned not to comfort with food and model healthy eating by eating healthy foods in front of the children during the food breaks at preschool. Also, they were encouraged to initiate fun games that get the children moving. The third meeting concerned the interaction with parents regarding a healthy lifestyle.

#### PLAYgrounds for TODdlers programme (PLAYTOD)

PLAYTOD was designed to coach ECEC teachers on how to stimulate physical activity (in particular fundamental movement skills) on the playgrounds of preschools. It focussed on the teachers’ knowledge and practices in order to create a challenging outdoor environment in which young children can practice their motor skills. A detailed description of the intervention was previously published [20, 23]. In short, 2 face-to-face training sessions of 2 hours each were organised for 4 groups of ECEC teachers. Each meeting was led by 2 certified PLAYTOD trainers. The first training session included theory about the importance of (outdoor) physical activity and a basic inviting structure of the playground (with the use of different activity zones) for variation in fundamental movement skills was demonstrated and practiced. After 2 weeks, ECEC teachers received a coaching on the job session. In the second training session, the activating role of the teachers (prompts) on the playground was practiced and reviewed in more detail. PLAYTOD was derived from the effective PLAYgrounds programme for primary schools [24, 25].

The adherence to the multi-component programmes was determined by attendance records. ECEC teachers who attended at least 2 meetings of AHS and 1 training session of PLAYTOD received certificates.

### Data collection procedures and outcome measures

ECEC teachers were measured at preschools and asked to fill in questionnaires at baseline, after AHS (at 4 months) and after PLAYTOD (at 9 months). Furthermore, they were requested to fill in 3-day food- and physical activity records at baseline and 9 months. Children were measured at preschools and parents were asked to fill in questionnaires and 3-day food- and physical activity records at baseline and 9 months.

All measurements were performed by trained research staff and students of the Amsterdam University of Applied Sciences using Standard Operation Procedures. The study team was coordinated by 3 unblinded researchers (SM/MJ/NT) and 1 blinded researcher (MTS). Questionnaires, records and measurement score forms were coded to protect the privacy of the participants. Data were processed in Microsoft Excel by double data-entry (not applicable for records). A detailed description of the data collection procedures and outcome measures was previously published [20].

#### ECEC teacher and child characteristics

General demographic characteristics were obtained via questionnaires. Dates of birth were collected. Age was calculated in years for ECEC teachers and in months for children. The participant’s country of birth as well as the country of birth of the participant’s mother and father were used to identify a migration background. First and second generation migration backgrounds were taken into account and included as ethnicity in our statistical models [26]. The highest level of education was subdivided in lower, intermediate or higher education groups [27].

#### ECEC teachers’ knowledge, attitude, practices, and level of confidence

Knowledge was assessed by 2 types of questions. Firstly, the teachers were asked to respond to 10 statements on the Dutch dietary guidelines [28]. Secondly, they had to indicate in 3 separate questions whether 5 food products (in total 15 food products) were ‘High’ or ‘Low’ in added sugars, salt and fibres, respectively [29]. A sum score for knowledge was calculated; each correctly answered question yielded 1 point with a maximum of 25 points.

Attitudes were evaluated through 5 individual statements regarding a healthy lifestyle. The statements were compiled by the research staff. A five-point Likert-scale was used. Answering options for the statements were totally disagree, slightly disagree, neutral, slightly agree and totally agree (1 to 5 points). The attitude statements related to different types of attitudes and were therefore separately assessed.

A modified version of the Child-care Food and Activity Practices Questionnaire (CFAPQ) [30] was used to assess food/activity-related practices. The following original CFAPQ scales were used: Food-related-Modelling/Encourage-balance-and-variety, Food-related-Teaching-about-Nutrition, Food-related-Pressure-to-Eat, Activity-related-Modelling, Activity-related-Psychological-Control, Activity-related-Teaching/Autonomy-Support and Activity-related-Going-Outdoors. Answering options for the questions were totally disagree/never, slightly disagree/rarely, neutral/sometimes, slightly agree/mostly and totally agree/always (1 to 5 points). A mean score per CFAPQ scale was calculated. Except for the scale Food-related-Teaching-about-Nutrition (Cronbach’s alpha 0.17), the scales showed comparable internal consistency to the original CFAPQ scales (Cronbach’s alpha ranging from 0.56–0.86). Because of its low internal consistency, the scale Food-related-Teaching-about-Nutrition was not included in further analysis. Furthermore, the scale Activity-related-Going-Outdoors was not included in further analysis as the questions in this scale turned out to be inapplicable for the context of the preschools.

The level of confidence related to promoting healthy eating and physical activity in young children was assessed using a confidence ruler [31]. The teachers indicated, on a scale of 1 (not confident at all) to 10 (extremely confident), how confident they were in supporting children and their parents in a healthy lifestyle for the children.

#### ECEC teachers’ and children’s BMI and body composition

Body weight (kg) was measured using a portable weighing scale (Seca 813) without children’s and teachers’ shoes or heavy clothing. Body height (cm) was measured with a portable stadiometer (Seca 213) without participants’ shoes. The measurements were performed (at least) twice to reduce measurement errors. Next, BMI (kg/m^2^) was calculated using the mean of the weight measurements and height measurements. For children, also BMI z-scores were assessed using World Health Organisation reference data (WHO Anthro). The weight status of the teachers was defined as underweight (BMI < 18.5), normal weight (18.5 ≥ BMI < 25.0), overweight (25.0 ≥ BMI < 30.0) and obesity (BMI ≥ 30.0). The weight status of children was evaluated by reference data of Cole et al. (2012) [32].

Total body resistance was measured in both the teachers and children using Bioelectrical Impedance Analysis (Bodystat 1500 MDD). A single prediction equation by Kyle et al. (2001) was used to calculate Fat Free Mass (FFM) in the teachers [33]. Thereafter, the Fat Mass (FM) in kg was calculated by subtracting the FFM from the total body weight. For children, a formula validated by de Beer et al. (2011) was used to calculate Total Body Water (TBW) [34]. Next, TBW and hydration constants discussed by Fomon et al. (1982) were used to calculate FFM [35]. The FM (kg) of children was calculated by subtracting the FFM from the total body weight. At last, the Fat Mass Index (FMI) and Fat Free Mass Index (FFMI) in kg/m^2^ were calculated for the teachers and children.

#### ECEC teachers’ and children’s dietary intake and physical activity level

ECEC teachers were asked to record their dietary intake per meal and physical activities per 0.5 h for 2 working days and 1 weekend day. Parents were asked to record their children’s dietary intake per meal and physical activities per 0.5 h during 2 week days and 1 weekend day [36]. For ECEC teachers, the self-reported intake of foods, were converted into energy and nutrient intake using the Dutch Food Composition Database 2016 [37] and national database for portion sizes [38]. Furthermore, MET-scores were assigned to the self-reported physical activities. The 2011 Compendium of Physical Activities was used as reference for MET-scores [39] and physical activity levels were calculated. For children, the recorded intake of foods and physical activities were not included in further analysis, as most of the parents did not fill in the records (or, at least not adequately).

### Sample size calculations

The sample size of 120 ECEC teachers was based on a medium effect size (Cohen’s d: 0.50), a 1-sided alpha of 5% and a power of 80%, taken into account a design effect (mean cluster size: 3, ICC: 0.05) and a 10% drop-out rate. The sample size calculations were conducted prior to the onset of the study [20]. It was chosen, a priori, to use a 1-sided alpha of 5% in the sample size calculations as it was aimed to examine whether the intervention improved the teachers’ knowledge, attitude, food/activity-related practices, and level of confidence in promoting healthy eating and physical activity (H0: β = 0 / H1: β > 0).

### Statistical analyses

Descriptive statistics (n (%) or mean (SD)) were used to describe the characteristics of the study population. Linear mixed model analyses were performed to determine the effects of the intervention.

In the analyses concerning ECEC teachers, participant and preschool location were added as random intercepts to take into account the repeated measures in the teachers and clustered data structure. Overall models with treatment (intervention or control group) and the baseline value of a specific outcome measure were made to assess the overall treatment effect of the intervention. Next, time and an interaction between treatment and time were added to the model to assess the effects of the intervention at 4 and 9 months. In addition, the models were adjusted for age (years), ethnicity and level of education of the teachers.

In the analyses concerning children, preschool location was added as random intercept to take into account the clustered data structure. Overall models with treatment (intervention or control group) and the baseline value of a specific outcome measure were made to assess the overall treatment effects of the intervention. In addition, the models were adjusted for sex, age (months) and ethnicity of the child.

Regression coefficients (difference), 1-sided p values and 1-sided 95% Confidence Limits (CL) were computed for all crude and adjusted mixed models. The statistical significance was set at p < 0.05 (1-sided). All statistical analyses were performed in IBM SPSS Statistics version 24 (IBM corp., Armonk, NY, USA) using the intention-to-treat principle (in which all participants who started the intervention were included in the analyses).

## Results

### Study sample

**Fig 2:**
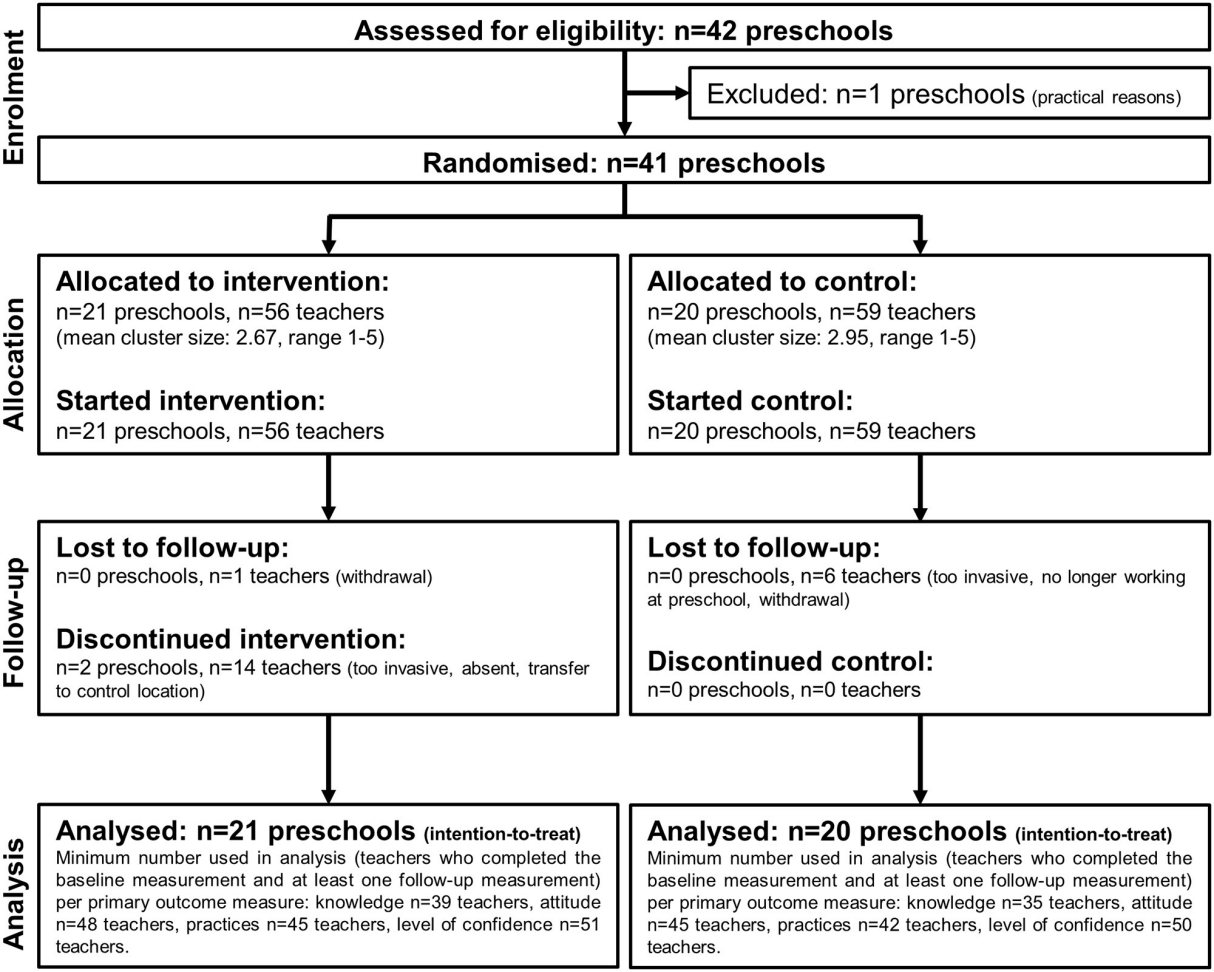
Flow diagram of the study sample.

The final sample included 41 preschools in which 115 ECEC teachers (71% of the 162 available teachers at the start of the study) and 249 children/parents were recruited. In total, 7 teachers dropped out of the study: 3 teachers in the control group indicated that the measurements were too time consuming/invasive, 2 teachers in the control group were no longer working at the participating childcare organisation, and 1 teacher in both the intervention- and control group withdrew their consent to participate in the study. Different numbers of ECEC teachers were used in analyses, as the teachers did not always complete the questionnaires. Fig 2 shows a flow diagram of the study sample.

### ECEC teacher and child characteristics

Table 1 shows the general characteristics of the teachers. All teachers were women and their mean age was 42 [9] years. Most of the teachers had a Dutch (38%) or Moroccan (33%) ethnicity. The percentages of overweight and obesity were, 30 and 35, respectively.

**Table 1:** General characteristics of the ECEC teachers.

	Total (n = 41 preschools, n = 115 teachers)	Intervention (n = 21 preschools, n = 56 teachers)	Control (n = 20 preschools, n = 59 teachers)
**Age** in years, mean (SD)	42 (9)	43 (10)	41 (9)
**Ethnicity**, n (%) ^a^^,^ ^b^			
Dutch	42 (38)	21 (39)	21 (38)
Moroccan	36 (33)	16 (30)	20 (36)
Turkish	8 (7)	5 (9)	3 (5)
Other western	12 (11)	5 (9)	7 (13)
Other western	12 (11)	7 (13)	5 (9)
**Level of Education**, n (%) ^c^			
Lower education	0 (0)	0 (0)	0 (0)
Intermediate education	79 (73)	38 (70)	41 (75)
Higher education	30 (28)	16 (30)	14 (26)
**Weight status**, n (%) ^d^			
Normal weight	37 (36)	19 (40)	18 (32)
Overweight	31 (30)	9 (19)	22 (39)
Obesity	36 (35)	19 (40)	17 (30)

Values are n (%) or mean (SD).

^a^ first and second generation migration backgrounds were taken into account.

^b^ 5 missing values.

^c^ 6 missing values.

^d^ 11 missing values.

Table 2 presents the general characteristics of the children. In total, 49% were girls. Their mean age was 3.0 (0.2) years. Most of the children (35%) had a Moroccan ethnicity. The percentages of overweight and obesity in children were, 13 and 3, respectively.

**Table 2:** General characteristics of the children.

	Total (n = 40 preschools, n = 249 children)	Intervention (n = 21 preschools, n = 137 children)	Control (n = 19 preschools, n = 112 children)
**Sex**, n (%) girls	121 (49)	70 (51)	51 (46)
**Age** in years, mean (SD)	3.0 (0.2)	3.0 (0.2)	3.0 (0.2)
**Ethnicity**, n (%) ^a^			
Dutch	42 (19)	22 (18)	20 (20)
Moroccan	78 (35)	45 (37)	33 (33)
Turkish	39 (18)	19 (16)	20 (20)
Other western	19 (9)	8 (7)	11 (11)
Other non-western	44 (20)	27 (22)	17 (17)
**Level of Education Respondent**, n (%) ^b^			
Lower education	42 (20)	27 (24)	15 (16)
Intermediate education	89 (43)	47 (42)	42 (44)
Higher education	78 (37)	39 (35)	39 (41)
**Weight status**, n (%) ^c^			
Underweight	19 (9)	11 (9)	8 (8)
Normal weight	171 (76)	90 (74)	81 (79)
Overweight	28 (13)	15 (12)	13 (13)
Obesity	6 (3)	5 (4)	1 (1)

Values are n (%) or mean (SD).

^a^ first and second generation migration backgrounds were taken into account. 27 missing values.

^b^ Respondent was in 98% of the cases a parent, 40 missing values.

^c^ 25 missing values.

### Intervention effects

Tables 3 and 4 show observed data. Tables 5 and 6 present modelled data. A positive effect of the intervention on ECEC teachers’ knowledge about the Dutch dietary guidelines was found after AHS (at 4 months) (difference = 1.38; 1-sided 95% CL = 0.29; p = 0.02). This effect was not sustained at 9 months. For the following attitude statements overall positive effects of the intervention were found: ‘I think it is important that I have a healthy lifestyle’, ‘I think education about healthy lifestyle to children belongs at preschool’ and ‘I think education about healthy lifestyle to parents belongs at preschool’. Furthermore, the intervention had an overall positive effect on the practice scale Activity-related-Modelling (difference = 0.16; 1-sided 95% CL = 0.06; p = 0.01). For the practice scale Activity-related-Teaching/Autonomy-Support, a positive effect was found after PLAYTOD (at 9 months) (difference = 0.23; 1-sided 95% CL = 0.09; p = 0.01). No effects of the intervention were found on the scale Activity-related-Psychological-Control and the food-related practice scales Modelling/Encourage-balance-and-variety and Pressure-to-Eat. In addition, no effects were found at the level of the teachers’ confidence in promoting healthy eating and physical activity.

**Table 3:** Observed data for ECEC teachers’ knowledge, attitude, practices, and level of confidence.

		Intervention						Control					
		Baseline		4 months		9 months		Baseline		4 months		9 months	
		N	Mean (SD)	N	Mean (SD)	N	Mean (SD)	N	Mean (SD)	N	Mean (SD)	N	Mean (SD)
**Knowledge**	Dietary Guidelines ^a^	39	17.90 (4.34)	30	20.07 (3.75)	32	19.72 (3.48)	35	18.63 (3.15)	33	19.27 (3.35)	28	19.39 (3.50)
**Attitude**	Statement 1 ^b^	49	3.73 (1.15)	48	3.92 (1.01)	42	4.07 (0.97)	46	3.80 (1.03)	45	3.98 (0.92)	39	4.05 (0.94)
	Statement 2 ^c^	49	4.10 (1.25)	48	4.54 (0.85)	42	4.52 (0.80)	45	4.29 (1.01)	44	4.20 (0.90)	38	4.18 (1.06)
	Statement 3 ^d^	50	4.04 (1.31)	48	4.31 (0.80)	43	4.37 (0.79)	45	4.02 (1.03)	43	4.05 (1.09)	38	4.11 (0.73)
	Statement 4 ^e^	48	3.75 (1.19)	46	4.09 (0.94)	42	3.95 (0.85)	46	3.83 (1.08)	45	3.78 (1.06)	40	3.58 (0.96)
	Statement 5 ^f^	49	3.86 (1.17)	47	4.26 (0.94)	43	4.12 (0.79)	46	3.91 (1.21)	44	3.86 (1.03)	40	3.42 (1.24)
**Practices**	Food-related-MOD-ENC ^g^	47	4.39 (0.79)	43	4.65 (0.40)	39	4.47 (0.66)	43	4.45 (0.46)	40	4.49 (0.51)	38	4.50 (0.47)
	Food-related-PE ^h^	48	2.77 (0.86)	46	2.25 (0.81)	39	2.14 (0.76)	46	2.43 (1.01)	43	2.31 (0.81)	37	2.23 (0.82)
	Activity-related-MOD ^i^	45	3.82 (0.46)	45	3.88 (0.40)	37	4.03 (0.39)	42	3.79 (0.42)	39	3.76 (0.35)	37	3.76 (0.39)
	Activity-related-PC ^j^	45	1.99 (0.48)	45	1.92 (0.39)	34	1.97 (0.39)	42	2.08 (0.37)	39	1.98 (0.36)	36	1.92 (0.38)
	Activity-related-TAS ^k^	46	3.52 (0.49)	43	3.63 (0.47)	39	3.70 (0.46)	43	3.47 (0.56)	37	3.58 (0.55)	37	3.44 (0.50)
	Activity-related Single Item ^l^	50	4.48 (0.81)	49	4.49 (0.74)	43	4.42 (0.70)	44	4.59 (0.50)	40	4.70 (0.56)	37	4.65 (0.54)
**Confidence**	Supporting children ^m^	51	7.63 (1.15)	49	7.74 (1.01)	50	7.79 (0.92)	50	7.53 (1.11)	47	7.69 (0.81)	47	7.65 (1.07)
	Supporting parents ^n^	51	7.27 (1.37)	49	7.34 (0.91)	50	7.35 (0.95)	50	6.79 (1.32)	47	7.16 (1.21)	47	7.20 (1.10)

^a^ Knowledge about the Dutch dietary guidelines.

^b^ Statement 1: I am someone who is conscious of healthy eating.

^c^ Statement 2: I think it is important that I have a healthy lifestyle.

^d^ Statement 3: I feel responsible for healthy nutrition and exercise patterns of children.

^e^ Statement 4: I think education about healthy lifestyle to children belongs at preschool.

^f^ Statement 5: I think education about healthy lifestyle to parents belongs at preschool.

^g^ Food-related-Modelling/Encourage-balance-and-variety.

^h^ Food-related-Pressure-to-Eat.

^i^ Activity-related-Modelling.

^j^ Activity-related-Psychological-Control.

^k^ Activity-related-Teaching/Autonomy-Support.

^l^ Activity-related Single Item: How often do you have outdoor toys available for the children.

^m^ Level of confidence in supporting children in pursuing a healthy lifestyle.

^n^ Level of confidence in supporting parents in a healthy lifestyle for their child.

**Table 4:** Observed data for ECEC teachers’ and children’s BMI (z-score) and body composition.

		Intervention						Control					
		Baseline		4 months		9 months		Baseline		4 months		9 months	
		N	Mean (SD)	N	Mean (SD)	N	Mean (SD)	N	Mean (SD)	N	Mean (SD)	N	Mean (SD)
**Teachers**	BMI (kg/m^2^)	43	27.59 (4.46)	42	27.76 (4.75)	41	27.43 (4.67)	52	27.74 (5.28)	50	27.56 (5.14)	49	27.74 (5.46)
	FMI (kg/m^2^)	43	11.17 (3.04)	42	11.04 (3.16)	40	10.95 (3.06)	52	11.14 (3.56)	50	10.91 (3.47)	48	11.11 (3.82)
	FFMI (kg/m^2^)	43	16.42 (1.64)	42	16.72 (1.79)	40	16.61 (1.82)	52	16.60 (1.93)	50	16.64 (1.90)	48	16.71 (1.85)
**Children**	BMI z-score	101	0.81 (0.95)	-	-	101	0.74 (1.08)	93	0.75 (0.80)	-	-	93	0.71 (0.87)
	BMI (kg/m^2^)	101	16.67 (1.38)	-	-	101	16.47 (1.61)	93	16.55 (1.14)	-	-	93	16.38 (1.23)
	FMI (kg/m^2^)	64	3.42 (1.23)	-	-	64	3.95 (1.31)	62	3.06 (1.07)	-	-	62	3.58 (1.30)
	FFMI (kg/m^2^)	64	13.44 (0.73)	-	-	64	12.72 (0.70)	62	13.46 (0.72)	-	-	62	12.86 (0.76)

BMI: Body Mass Index. FMI: Fat Mass Index. FFMI: Fat Free Mass Index.

**Table 5:** Mixed models for ECEC teachers’ knowledge, attitude, practices, and level of confidence.

		Crude*				Adjusted**	
		difference	1-sided 95% CL	*P*	difference	1-sided 95% CL	*P*
**Knowledge** ^a^	Overall	0.86	-0.01	0.05	1.18	0.32	0.01
	4 months	1.38	0.29	0.02	1.65	0.56	0.01
	9 months	0.34	-0.76	0.31	0.71	-0.40	0.15
**Attitude**							
Statement 1 ^b^	Overall	-0.01	-0.26	0.52	0.01	-0.23	0.47
	4 months	-0.06	-0.36	0.63	-0.06	-0.34	0.63
	9 months	0.06	-0.26	0.38	0.09	-0.21	0.31
Statement 2 ^c^	Overall	0.37	0.14	0.00	0.39	0.17	0.00
	4 months	0.37	0.07	0.02	0.38	0.08	0.02
	9 months	0.36	0.04	0.03	0.41	0.09	0.02
Statement 3 ^d^	Overall	0.25	-0.05	0.08	0.28	-0.00	0.05
	4 months	0.24	-0.09	0.12	0.27	-0.06	0.09
	9 months	0.26	-0.09	0.11	0.29	-0.04	0.08
Statement 4 ^e^	Overall	0.34	0.09	0.02	0.35	0.11	0.01
	4 months	0.33	0.02	0.04	0.37	0.07	0.02
	9 months	0.36	0.04	0.04	0.33	0.02	0.04
Statement 5 ^f^	Overall	0.55	0.31	0.00	0.53	0.28	0.00
	4 months	0.43	0.12	0.01	0.43	0.12	0.01
	9 months	0.69	0.37	0.00	0.64	0.31	0.00
**Practices**							
Food-related-MOD-ENC ^g^	Overall	0.06	-0.10	0.27	0.05	-0.11	0.31
	4 months	0.15	-0.03	0.09	0.13	-0.05	0.12
	9 months	-0.04	-0.23	0.65	-0.05	-0.23	0.67
Food-related-PE ^h^	Overall	-0.24	0.03	0.07	-0.22	0.04	0.08
	4 months	-0.22	0.07	0.10	-0.24	0.05	0.08
	9 months	-0.26	0.04	0.08	-0.20	0.10	0.14
Activity-related-MOD ^i^	Overall	0.16	0.06	0.01	0.13	0.05	0.01
	4 months	0.12	0.00	0.05	0.10	0.00	0.05
	9 months	0.22	0.10	0.00	0.18	0.07	0.00
Activity-related-PC ^j^	Overall	0.03	0.15	0.70	0.00	0.12	0.51
	4 months	-0.01	0.11	0.43	-0.05	0.07	0.24
	9 months	0.09	0.22	0.89	0.07	0.20	0.81
Activity-related-TAS ^k^	Overall	0.11	-0.02	0.08	0.09	-0.03	0.10
	4 months	-0.01	-0.15	0.54	-0.02	-0.16	0.57
	9 months	0.23	0.09	0.01	0.20	0.06	0.01
Activity-related	Overall	-0.18	-0.37	0.95	-0.20	-0.36	0.98
Single Item ^l^	4 months	-0.17	-0.40	0.90	-0.21	-0.41	0.95
	9 months	-0.20	-0.43	0.92	-0.19	-0.40	0.93
**Confidence**							
Supporting children ^m^	Overall	0.10	-0.19	0.28	0.13	-0.17	0.24
	4 months	0.07	-0.28	0.37	0.11	-0.24	0.30
	9 months	0.14	-0.20	0.25	0.14	-0.20	0.25
Supporting parents ^n^	Overall	0.00	-0.29	0.49	-0.05	-0.32	0.63
	4 months	0.05	-0.29	0.41	0.02	-0.30	0.46
	9 months	-0.04	-0.38	0.57	-0.12	-0.45	0.74

* Adjusted for the baseline value of the outcome variable.

** Adjusted for the baseline value of the outcome variable, age, ethnicity and level of education.

^a^ Knowledge about the Dutch dietary guidelines.

^b^ Statement 1: I am someone who is conscious of healthy eating.

^c^ Statement 2: I think it is important that I have a healthy lifestyle.

^d^ Statement 3: I feel responsible for healthy nutrition and exercise patterns of children.

^e^ Statement 4: I think education about healthy lifestyle to children belongs at preschool.

^f^ Statement 5: I think education about healthy lifestyle to parents belongs at preschool.

^g^ Food-related-Modelling/Encourage-balance-and-variety.

^h^ Food-related-Pressure-to-Eat (a negative estimate is in favour of the intervention group).

^i^ Activity-related-Modelling.

^j^ Activity-related-Psychological-Control (a negative estimate is in favour of the intervention group.

^k^ Activity-related-Teaching/Autonomy-Support.

^l^ Activity-related Single Item: How often do you have outdoor toys available for the children.

^m^ Level of confidence in supporting children in pursuing a healthy lifestyle.

^n^ Level of confidence in supporting parents in a healthy lifestyle for their child.

**Table 6:** Mixed models for ECEC teachers’ and children’s BMI (z-score) and body composition.

		Crude*			Adjusted**		
		difference	1-sided 95% CL	*P*	difference	1-sided 95% CL	*P*
**ECEC teachers**							
BMI (kg/m^2^)	Overall	0.15	0.43	0.82	0.24	0.53	0.92
	4 months	0.10	0.40	0.72	0.19	0.50	0.84
	9 months	0.20	0.50	0.87	0.30	0.61	0.94
FMI (kg/m^2^)	Overall	-0.05	0.22	0.38	0.02	0.30	0.55
	4 months	-0.09	0.19	0.29	-0.04	0.26	0.41
	9 months	0.01	0.29	0.51	0.10	0.39	0.70
FFMI (kg/m^2^)	Overall	0.21	0.01	0.04	0.22	0.03	0.03
	4 months	0.20	-0.01	0.05	0.22	0.02	0.04
	9 months	0.21	0.00	0.05	0.22	0.01	0.04
**Children**							
BMI z-score	Overall	-0.03	0.10	0.36	-0.06	0.07	0.21
BMI (kg/m^2^)	Overall	-0.02	0.18	0.44	-0.07	0.12	0.27
FMI (kg/m^2^)	Overall	-0.01	0.25	0.47	-0.12	0.14	0.21
FFMI (kg/m^2^)	Overall	-0.08	-0.27	0.75	-0.05	-0.24	0.65

* Adjusted for the baseline value of the outcome variable.

** For ECEC teachers, adjusted for the baseline value of the outcome variable, age, ethnicity and level of education.

For children, adjusted for the baseline value of the outcome variable, sex, age and ethnicity. BMI: Body Mass Index. FMI: Fat Mass Index. FFMI: Fat Free Mass Index.

For ECEC teachers’ and children’s BMI (z-score) no effects of the intervention were found. An overall positive effect was observed on teachers’ FFMI. No effects of the intervention were observed on dietary intake and physical activity levels of ECEC teachers.

## Discussion

This cluster randomised controlled trial showed positive effects on ECEC teachers’ knowledge about the Dutch dietary guidelines, attitudes towards a healthy lifestyle and some activity-related practices. No effects were seen on the teachers’ food-related practices and the level of confidence in promoting healthy eating and physical activity in young children.

An important finding of our study was the positive effect of the intervention on the teachers’ knowledge. After AHS, a mean increase of 2 points (on a maximum knowledge score of 25 points) was observed in the intervention group. Also other studies with interventions for ECEC teachers report positive effects on the teachers’ knowledge about nutrition and physical activity [40, 41]. However, in these studies no retention measurements were carried out. Our study provides additional information, we observed that the positive effect of AHS did not sustain and believe that repetition and elaboration strategies are needed to improve retention. For example, the coaches who led the AHS meetings could organise a recurring meeting every few months. In these meetings, the dietary guidelines can be further discussed and questions or recent experiences from daily practice can be addressed. We have noticed that teachers really enjoyed sharing experiences and learning new knowledge during the programmes.

We found positive effects of the intervention on 3 of the 5 attitude statements regarding a healthy lifestyle. In the intervention group, the scores for each attitude statement improved after the programme AHS. This was not the case for the control group. For the statement ‘I think it is important that I have a healthy lifestyle’, a significant difference with the control group was found. This suggests that ECEC teachers in the intervention group were, after the intervention, more aware of their role in setting a healthy example. For the 2 individual statements regarding education about healthy lifestyle at preschools also a significant effect of the intervention was found. These results indicate that ECEC teachers were, after the intervention, more open to initiatives regarding a healthy lifestyle at preschools compared to the control group. For the statements ‘I am someone who is conscious of healthy eating’ and ‘I feel responsible for healthy nutrition and exercise patterns of children’, no significant effects of the intervention were observed. Particularly for the last of these 2 statements, the mean score at baseline was already high in both groups, so there was not much room left for improvement. All ECEC teachers from our study seem to acknowledge their responsibility related to a healthy lifestyle. Hardy et al. (2010) conducted a comparable study among Australian preschools and did not find significant changes in the teacher’s attitudes relating to healthy eating and physical activity [42]. The authors describe that the low-intensity and short implementation period (20 weeks) of their programme could explain their findings. Our intervention suggests a more positive outcome, taking into account the positive effects for some attitudinal measures.

No effects of the intervention were seen on food-related practices. The healthy food policy of the participating childcare organisation may have affected this outcome. Children only received fruit and vegetables during the short food breaks at preschool and were only allowed to drink water.

The overall intervention had a positive effect on the practice scale Activity-related-Modelling. This practice scale includes questions like ‘How often do you set an example for the children by being physically active in front of them?’. After PLAYTOD, also a positive effect was found on the practice scale Activity-related-Teaching/Autonomy-Support. This practice scale includes questions like ‘How often do you say positive things to motivate children to be more active?’. We believe the positive results can be attributed to the fact that, in the PLAYTOD training sessions, the teachers had the possibility to practice their role on the playground. With a coaching on the job session, the teachers received specific instructions for improvement. Observations on playgrounds also showed that, after PLAYTOD, the observed role of ECEC teachers on intervention playgrounds was more active. The results of the observations on playgrounds were recently published [23].

In total, 30% of the teachers had overweight and 35% had obesity. The percentage of obesity in our sample is high compared to the national average of women with obesity in the Netherlands (16.1% in 2019) [43]. No effects of the intervention were found on the teachers’ personal dietary intake, physical activity level and BMI. This may be explained by the fact that in only 1 meeting of AHS attention was devoted to the teachers’ personal lifestyle outside preschool.

No effects of the intervention were found on BMI z-scores and body composition of children. We hypothesised that changes in ECEC teachers may eventually transfer to changes in children. Egert et al (2018) show, in their meta-analysis on the impact of in-service ECEC teacher training on child outcomes, that quality improvements can lead to small positive effects on child development [44]. More time might be needed before small changes in children’s eating and physical activity behaviours may be observed, and finally, may result in weight-related changes. Similar to our study, van de Kolk et al. (2019) did not find effects of their obesity prevention intervention on BMI z-scores in Dutch preschool aged children [45]. They also suggest that their follow-up period was not long enough or changes caused by their intervention were not substantial enough to result in an effect on BMI z-scores. In a recent systematic review, on childcare-based interventions with direct parental involvement, the authors describe promising effects of combined programmes on children’s energy balance-related behaviours. However, evidence on effectiveness is limited, particularly for weight-related outcomes [46]. After our study, we asked in short interviews what the teachers think is still needed to promote healthy behaviours. The majority of ECEC teachers emphasised that a higher level of parental engagement is necessary. We therefore think it is important in the future to focus more on the collaboration between ECEC teachers and parents concerning lifestyle behaviours of children.

Strengths of this study include the design and multi-component character of the intervention. Moreover, the diverse and relevant study population is a strength, taken into account the high prevalence of overweight and obesity in deprived urban areas. However, the study is limited in the ability to generalise the findings, as only preschools of 1 childcare organisation were included. In addition, the primary outcomes were assessed via questionnaire. Methods for data collection that are less prone to socially desirable responding (e.g. repeated observations) may therefore be of use in future trials. As multiple comparisons were made, there is a possibility of false discoveries. When a Benjamini and Hochberg [47] corrected significance level based on 14 multiple comparisons is applied, we still find a positive effect on 2 of the 5 attitude statements and the practice scale Activity-related-Modelling.

## Conclusions

Our preschool-based intervention contributed to the professional development of ECEC teachers. It had positive effects on the teachers’ knowledge about the Dutch dietary guidelines, attitudes towards a healthy lifestyle and some activity-related practices. No effects were found on the teachers’ food-related practices and level of confidence in promoting healthy eating and physical activity. This study addresses the call for early interventions to prevent overweight and obesity and might contribute to minimising health inequalities in young children.

## Supporting information

S1 File CONSORT checklist. (DOCX)

S2 File Protocol. (PDF)

S3 File Translation protocol. (PDF)
