# Supplementary material for: The effects of a preschool-based intervention for Early Childhood Education and Care teachers in promoting healthy eating and physical activity in young children: A cluster randomised controlled trial
Source: PLoS One. 2021 Jul 23;16(7):e0255023. doi: 10.1371/journal.pone.0255023 (PMC8302250; doi:10.1371/journal.pone.0255023)
Supplement: S2 File — (PDF) [file pone.0255023.s002.pdf]

Onderzoeksprotocol

Algemene gegevens

**Titel**	PreSchool@HealthyWeight; Op weg naar een gezonde kinderopvang voor elke peuter
**Datum**	01-07-2016
**Versienummer**	1
**Indiener**	Dr. Ir. Peter J.M. Weijs<br>Coördinator onderzoek / Sectie Voeding en Diëtetiek<br>Afdeling interne geneeskunde<br>VUmc Amsterdam<br>De Boelelaan 1117, ZH4A12<br>1081 HV Amsterdam<br>p.weijs@vumc.nl<br><br>Lector Gewichtsmanagement<br>Hogeschool van Amsterdam<br>Faculteit Bewegen, Sport en Voeding<br>Kenniscentrum Bewegen, Sport en Voeding<br>Dr. Meurerlaan 8, 1067 SM Amsterdam<br>p.j.m.weijs@hva.nl
**Coördinerend onderzoeker**	Dr. Ir. Martinette Streppel<br>Senior onderzoeker<br>Hogeschool van Amsterdam<br>Faculteit Bewegen, Sport en Voeding<br>Dr. Meurerlaan 8, 1067 SM Amsterdam<br>m.t.streppel@hva.nl
**Hoofdonderzoeker(s)**	Dr. Ir. Peter J.M. Weijs
**Opdrachtgever (verrichter)**	Hogeschool van Amsterdam (HvA)<br>Hogeschool van Amsterdam

Onderzoekgegevens

**Rationale**	De prevalentie van overgewicht en obesitas bij kinderen in Nederland is hoog (Schönbeck *et al*., 2011). Het Centraal Bureau voor de Statistiek laat op basis van zelfgerapporteerde waarden zien dat in 2015 bij 12,2% van de kinderen tussen de 2 en 9 jaar sprake was van overgewicht (Centraal Bureau voor de Statistiek, 2016). Zowel in de kinderjaren als op latere leeftijd vergroten overgewicht en obesitas de kans op gezondheidsproblemen (Volkgezondheidenzorg.info, 2016).<br>Het is bekend dat (ernstig) overgewicht samenhangt met dagelijkse keuzes in voedings- en beweeggedrag en dat deze gedragingen verschillen tussen etnische en sociaaleconomische groepen (de Munter, van Valkengoed, Agyemang, Kunst, & Stronks, 2010; Nicolaou, Nierkens, & Middelkoop, 2013). Al op zeer jonge leeftijd zijn verschillen in Body Mass Index (BMI) tussen sociaaleconomische groepen aanwezig (Bouthoorn *et al*., 2014). Resultaten van de Vijfde Landelijke Groeistudie laten zien dat het percentage kinderen met overgewicht en obesitas hoger is bij kinderen met een Turkse of Marokkaanse afkomst dan bij Nederlandse kinderen (Schönbeck, van Buuren, 2010).<br>Het leeftijdsinterval van 2 tot 6 jaar is belangrijk voor de ontwikkeling van overgewicht bij volwassenen (de Kroon, Renders, van Wouwe, van Buuren, & Hirasing, 2010). Bovendien speelt de omgeving waarin een kind opgroeit een relevante rol bij het ontstaan van (ernstig) overgewicht. Naast de thuissituatie is de kinderopvang een invloedrijke omgevingsfactor (Gubbels *et al*., 2010).<br>Op voorscholen werken Pedagogisch Medewerkers (PMers) met kinderen tussen de 2,5 en 4 jaar. PMers zijn getraind in het ondersteunen van de ontwikkeling en opvoeding van kinderen. De medewerkers kunnen invloed hebben op wat kinderen eten en drinken en hoeveel ze bewegen in de voorschoolse periode. Echter, PMers zijn niet opgeleid om begeleiding te geven op het gebied van leefstijl. Er is sprake van handelingsverlegenheid voortkomend uit een gebrek aan kennis en aan interactievaardigheden met ouders/verzorgers omtrent dit onderwerp.<br>In opdracht van het Ministerie van Volksgezondheid, Welzijn en Sport is door het Voedingscentrum, Nederlands Jeugdinstituut, Nederlands Instituut voor Sport en Bewegen, TNO, VeiligheidNL en Pharos, vanuit het maatschappelijke belang om kinderen vanaf heel jonge leeftijd zo

	gezond mogelijk te laten opgroeien, een training voor PMers ontwikkeld: Een Gezonde Start 2.0 (EGS2). EGS2 leert PMers hoe zij samen met ouders/verzorgers een gezonde, actieve en veilige omgeving kunnen bieden aan kinderen en hoe zij zelf het gezonde voorbeeld kunnen geven. Het landelijke programma is primair gericht op interactievaardigheden met ouders/verzorgers.<br>Met een aangepaste versie van de aangetoond effectieve PLAYgrounds interventie wordt, door een uitnodigende en gestructureerde inrichting van de buitenruimte en stimulans van het actief gebruik van de buitenruimte, lichamelijke beweging van kinderen gestimuleerd (Janssen, Toussaint, van Willem, & Verhagen, 2011; Janssen, Twisk, Toussaint, van Mechelen, & Verhagen, 2015). Middels instructie van een trainer krijgt een PMer handvatten aangereikt om aan de slag te gaan met het beweeggedrag van kinderen in de buitenruimte.<br>PreSchool@HealthyWeight onderzoekt wat de PMer in de voorschool nodig heeft aan kennis, attitude en vaardigheden om een goede ondersteuning te kunnen geven aan kinderen en hun ouders/verzorgers bij een gezonde leefstijl. Met de bestaande training EGS2 en een aangepaste versie van de aangetoond effectieve PLAYgrounds training beoogt het onderzoek het vertrouwen van PMers in het geven van goede ondersteuning aan kinderen en hun ouders/verzorgers bij een gezonde leefstijl te vergroten. Er wordt geambieerd een bijdrage te leveren aan het bevorderen van een gezonde (gewichts)ontwikkeling van kinderen en het terugdringen van gezondheidsverschillen tussen kinderen met diverse sociaal-economische en cultureel-etnische achtergronden.<br>Het onderzoek wordt grotendeels uitgevoerd op voorscholen in Amsterdam Nieuw-West. In Nieuw-West is het probleem van overgewicht en obesitas groter dan in andere stadsdelen, dit hangt sterk samen met het lage opleidingsniveau, de lage inkomens en etniciteit van inwoners in de wijk (Gemeente Amsterdam, 2015).
**Doel**	Het primaire doel van de studie is het vergroten van het vertrouwen van PMers, werkzaam bij voorscholen van kinderopvangorganisatie Impuls in Amsterdam Nieuw-West, in het goed kunnen ondersteunen van kinderen en hun ouders/verzorgers bij een gezonde leefstijl.

	De secundaire doelstelling is het meten van het effect van het trainen van PMers in het geven van een goede ondersteuning t.a.v. een gezonde leefstijl op:<br>- kennis, attitude en vaardigheden van PMers en ouders/verzorgers t.a.v. een gezonde leefstijl;<br>- lichaamssamenstelling en BMI van PMers en kinderen;<br>- voedings- en beweeggedrag van PMers en kinderen.
**Studie design**	Het onderzoek betreft een cluster gerandomiseerde interventie studie. Voor de studie worden voorschool-locaties van kinderopvangorganisatie Impuls willekeurig toegewezen aan de interventie- of controlegroep.
**Studie populatie**	De studiepopulatie bestaat uit PMers, kinderen en hun ouders/verzorgers van voorscholen van kinderopvangorganisatie Impuls in Amsterdam Nieuw-West.
**Inclusie criteria**	- PMers met een vast dienstverband bij kinderopvangorganisatie Impuls in Amsterdam Nieuw-West.<br>- PMers met een leeftijd ≥ 18 jaar.<br>- PMers die ≥ de helft van hun dienstverband werken bij een voorschool-locatie van kinderopvangorganisatie Impuls in Amsterdam Nieuw-West.<br>- Kinderen met een leeftijd ≥ 2,5 jaar en ≤ 3 jaar.<br>- Kinderen en hun ouders/verzorgers die begeleiding krijgen van een geïncludeerde PMer op een voorschool-locatie van kinderopvangorganisatie Impuls in Amsterdam Nieuw-West.
**Exclusie criteria**	- PMers werkzaam bij meerdere voorschool-locaties (zowel een interventie- als een controlelocatie) van kinderopvangorganisatie Impuls.<br>- Voorschool-locaties waar al interventies plaatsvinden met betrekking tot een gezonde leefstijl waarvan de onderzoekers verwachten dat ze interveniëren met EGS2 en PLAYgrounds.
**Aantal proefpersonen/ sample grootte**	Voor het onderzoek worden voorschool-locaties van kinderopvangorganisatie Impuls willekeurig toegewezen aan de interventie- of controlegroep. Om een antwoord te geven op de hoofdvraag van het onderzoek worden 120 PMers geïncludeerd (60 PMers in de interventiegroep en 60 PMers in de controlegroep). De sample grootte is gebaseerd op een medium effect grootte (Cohen's d: 0.50), een 1-zijdige alpha van 5%, en een power van 80%. Daarnaast is er gecorrigeerd voor

	het design effect (gemiddelde cluster grootte: 3, ICC: 0.05) en rekening gehouden met 10% uitval.<br>Om antwoord te geven op de secundaire onderzoeksvragen worden per PMer 2 kinderen en hun ouders/verzorgers geïncludeerd. In totaal gaat het om 240 kinderen en hun ouders/verzorgers (120 kinderen en hun ouders/verzorgers in de interventiegroep en 120 kinderen en hun ouders/verzorgers in de controlegroep). Met 120 kinderen per groep is het mogelijk om, met een 2-zijdige alpha van 5% en een power van 80%, een klein tot medium effect grootte (Cohen's d: 0.35-0.40) aan te tonen. Dit beoogde aantal kinderen is vergelijkbaar met de sample grootte van een gecombineerde voedings- en beweeginterventie waar na 7 maanden follow-up een significant verschil in BMI z-score werd aangetoond tussen de interventie- en controlegroep (Alkon *et al.*, 2013)
**Werving proefpersonen**	Alle geschikte PMers van een geïncludeerde voorschool-locatie worden via een wijkmanager en/of coach van kinderopvangorganisatie Impuls (leidinggevende) mondeling ingelicht over het onderzoek. Daarnaast ontvangen PMers een informatiebrief met uitgebreide uitleg betreffende PreSchool@HealthyWeight. Vervolgens bezoeken onderzoekers van de HvA voorschool-locaties om mondeling toelichting te geven en eventuele vragen te beantwoorden. Voorafgaand aan de metingen wordt schriftelijk informed consent verkregen.<br>Ouders/verzorgers worden via PMers mondeling op de hoogte gebracht van het onderzoek. Daarnaast zal er via een nieuwsbrief van Impuls informatie worden verstrekt aan ouders/verzorgers. Bij de voorscholen van kinderopvangorganisatie Impuls bestaat de afspraak dat ouders/verzorgers van de kinderen minimaal 15 minuten aanwezig zijn op de voorschool. In deze 15 minuten zal door onderzoekers van de HvA mondeling en schriftelijk (via een brief) uitgebreide informatie over het onderzoek worden gegeven. Bij een volgend bezoek kunnen eventuele vragen worden beantwoord en wordt schriftelijk informed consent verkregen.
**Interventie**	PreSchool@HealthyWeight betreft onderzoek bij 3 doelgroepen: PMers, kinderen en hun ouders/verzorgers. Voor PMers bestaat het onderzoek uit een nulmeting, tussenmeting en nameting. Bij kinderen en hun ouders/verzorgers wordt alleen een nul- en nameting uitgevoerd.

	Na de nulmeting bij PMers, kinderen en hun ouders/verzorgers, starten PMers op een interventielocatie met het programma EGS2. De training bestaat uit drie bijeenkomsten en gaat aan de hand van theorie en opdrachten in op een gezonde leefstijl van PMers, kinderen en hun ouders/verzorgers. Bijeenkomst 1 is gericht op een gezonde leefstijl van kinderen en PMers zelf. In bijeenkomst 2 wordt aandacht besteed aan de interactie met kinderen rondom een gezonde leefstijl. De laatste bijeenkomst gaat over de interactie met ouders/verzorgers t.a.v. een gezonde leefstijl van hun kind(eren). De training begint met een instaptoets voor het toetsten van initieel kennisniveau van PMers en wordt afgesloten met een eindtoets. Aansluitend op het programma vindt een periode plaats (± 3 maanden) waarin PMers verkregen kennis en vaardigheden t.a.v. een gezonde leefstijl in de praktijk brengen. PMers krijgen een actieve rol door feedback te geven aan ouders/verzorgers op het voedings- en beweeggedrag van hun kind(eren). Deze feedback is gebaseerd op een uitgebreide analyse van het voedings- en beweeggedrag van de kinderen (gegevens verzameld bij de nulmeting van kinderen en hun ouders/verzorgers) en wordt uitgevoerd door onderzoekers en studenten van de HvA.<br>Om het effect van de training EGS2 te meten wordt een tussenmeting verricht bij PMers. Hierbij zullen de resultaten van de instap- en eindtoets van EGS2 worden meegenomen. Na de tussenmetingen volgt een periode (± 3 maanden) waarin een aangepaste versie van de aangetoond effectieve PLAYgrounds training wordt uitgevoerd. Middels instructie door een trainer gaan de PMers aan de slag met beweeggedrag van de kinderen in de buitenruimte.<br>Op voorschool-locaties in de controlegroep worden de trainingen EGS2 en PLAYgrounds niet aangeboden en krijgen ouders/verzorgers geen feedback op het voedings- en beweeggedrag van hun kind(eren). Met een nameting bij PMers, kinderen en hun ouders/verzorgers wordt de dataverzameling van het onderzoek afgesloten.<br><br>Naast PreSchool@HealthyWeight zullen de kinderen, als Sarphati-cohort, onderdeel uitmaken van het Sarphati Amsterdam (Sarphati Amsterdam, 2016). Hiermee is het mogelijk om effecten op langere termijn vast te stellen.

	De nul- en nameting voor PMers bestaat uit:<br>- Het invullen van een vragenlijst (Gubbels, Sleddens, Raaijmakers, Gies, & Kremers, 2015) gericht op kennis, attitude en vaardigheden t.a.v. een gezonde leefstijl. In de vragenlijst komt het vertrouwen van PMers in het goed kunnen ondersteunen van kinderen en hun ouders/verzorgers bij een gezonde leefstijl aan bod. Tevens worden bij de nulmeting in deze vragenlijst enkele demografische kenmerken nagevraagd.<br>- Het meten van lengte en gewicht (voor het berekenen van de BMI).<br>- Het meten van lichaamssamenstelling middels Bio-elektrische Impedantie Analyse (BIA).<br>- Het invullen van een 3 daags eetdagboek.<br>- Het invullen van een 3 daags beweegdagboek.<br>- Het meten van fysieke activiteit op 7 dagen met een Physical Activity Monitor (PAM).<br>Getrainde studenten van de HvA meten lengte, gewicht en lichaamssamenstelling van een PMer op de voorschool-locatie zelf. Na de metingen wordt op papier een vragenlijst en eet- en beweegdagboek met bijhorende informatie verstrekt. Daarnaast wordt een PAM meegegeven. Na een week haalt een student de vragenlijst, de PAM, het eetdagboek en het beweegdagboek op bij de voorschool-locatie. Hierbij controleert de student de ingevulde vragenlijsten op volledigheid.<br><br>De tussenmeting voor PMers bestaat uit:<br>- Het invullen van een vragenlijst (Gubbels, Sleddens, Raaijmakers, Gies, & Kremers, 2015) gericht op kennis, attitude en vaardigheden t.a.v. een gezonde leefstijl . In de vragenlijst komt het vertrouwen van PMers in het goed kunnen ondersteunen van kinderen en hun ouders/verzorgers bij een gezonde leefstijl aan bod.<br>- Het meten van lengte en gewicht (voor het berekenen van de BMI).<br>- Het meten van lichaamssamenstelling middels BIA.<br>Getrainde studenten van de HvA meten lengte, gewicht en lichaamssamenstelling van een PMer op de voorschool-locatie zelf. Na de metingen wordt op papier een vragenlijst met bijhorende informatie verstrekt. Na een week haalt een student de vragenlijst op bij de

	voorschool-locatie. Hierbij controleert de student de ingevulde vragenlijsten op volledigheid.<br><br>De nul- en nameting voor kinderen bestaat uit:<br>- Het meten van lengte en gewicht (voor het berekenen van de BMI).<br>- Het meten van lichaamssamenstelling middels BIA.<br>- Een observatie van beweeggedrag (Schulz, Henderson, Sugden, & Barnett, 2011; McKenzie, Marshall, Sallis, & Conway, 2000).<br>De metingen en observaties vinden plaats op de voorschool-locatie zelf en worden uitgevoerd door getrainde studenten van de HvA.<br><br>De nul- en nameting voor ouders/verzorgers bestaat uit:<br>- Het invullen van een 3 daags eetdagboek van de kinderen.<br>- Het invullen van een 3 daags beweegdagboek van de kinderen.<br>- Een voedselfrequentievragenlijst (Dutman *et al*., 2011) van de kinderen (de vragenlijst wordt mondeling afgenomen door studenten van de HvA). Naast het eetdagboek (gericht op dag tot dag variatie in voedingsinname), geeft de gevalideerde voedselfrequentievragenlijst aanvullende informatie over voedingspatronen van de kinderen. De verkregen informatie wordt gebruikt door onderzoekers en studenten van de HvA voor het opstellen van feedback aan ouders/verzorgers. De voedselfrequentievragenlijst wordt alleen bij de nulmeting afgenomen.<br>- Een vragenlijst (Musher-Eizenman, Holub, 2007; O'Connor *et al*., 2014) gericht op kennis, attitude, vaardigheden en ouderbetrokkenheid t.a.v. een gezonde leefstijl van het kind. Daarnaast worden bij de nulmeting in deze vragenlijst enkele demografische kenmerken nagevraagd (de vragenlijst wordt mondeling afgenomen door studenten van de HvA).<br>Bij kinderopvangorganisatie Impuls bestaat de afspraak dat ouders/verzorgers minimaal 15 minuten aanwezig zijn op de voorschool. Deze tijd kan gebruikt worden voor het afnemen van de vragenlijsten. Tevens wordt op papier het eet- en beweegdagboek met bijhorende informatie verstrekt. Na een week haalt een student het eetdagboek en het beweegdagboek op bij de voorschool-locatie. Hierbij controleert de student de ingevulde dagboeken op volledigheid.

**Studie eindpunten**	Het vertrouwen van PMers in het goed kunnen ondersteunen van kinderen en hun ouders/verzorgers bij een gezonde leefstijl op een 10-puntsschaal.
**Studie parameters**	De volgende secundaire variabelen worden meegenomen in PreSchool@HealthyWeight:<br><br>**PMers**<br>- BMI (kg/m^2)<br>- Vetmassa (kg)<br>- Vetvrijemassa (kg)<br>- Energie (kcal)<br>- Eiwit (g)<br>- Vet (g)<br>- Verzadigd vet (g)<br>- Enkelvoudig onverzadigd vet (g)<br>- Meervoudig onverzadigd vet (g)<br>- Transvet (g)<br>- Koolhydraten (g)<br>- Vezels (g)<br>- Physical Activity Level<br>- Nederlandse Norm Gezond Bewegen<br>- Kennis, attitude en vaardigheden t.a.v. een gezonde leefstijl<br><br>**Kinderen**<br>- BMI (kg/m^2)<br>- Vetmassa (kg)<br>- Vetvrijemassa (kg)<br>- Energie (kcal)<br>- Eiwit (g)<br>- Vet (g)<br>- Verzadigd vet (g)<br>- Enkelvoudig onverzadigd vet (g)<br>- Meervoudig onverzadigd vet (g)<br>- Transvet (g)<br>- Koolhydraten (g)<br>- Vezels (g)<br>- Voedingspatronen

	- Physical Activity Level<br>- Nederlandse Norm Gezond Bewegen<br>- Motorische ontwikkeling<br><br>**Ouders/verzorgers**<br>- Kennis, attitude, vaardigheden en ouderbetrokkenheid t.a.v. een gezonde leefstijl
**Statistische analyses**	De statistische analyses worden uitgevoerd met de software IBM SPSS Statistics 22.0. Baseline-karakteristieken worden weergegeven met beschrijvende statistiek. Een ongepaarde t-toets of Mann-Whitney U toets wordt gebruikt om de baseline-karakteristieken van continue variabelen tussen de interventie- en controle groep te vergelijken. Voor categorische variabelen wordt de Chi-kwadraat toets of een Fisher's exact toets uitgevoerd.<br>Veranderingen over tijd binnen en tussen de groepen worden geanalyseerd met multi-level analyse, waarbij rekening wordt gehouden met de afhankelijkheid van de metingen binnen een voorschool-locatie.
**Belasting voor de proefpersoon**	**PMers**<br>- 3 x invullen van een vragenlijst gericht op kennis, attitude en vaardigheden t.a.v. een gezonde leefstijl (30 minuten per keer).<br>- 3 x meten van lengte, gewicht en lichaamssamenstelling (15 minuten per keer op voorschool-locatie, geen reistijd).<br>- 2 x 3 dagen bijhouden van een eetdagboek.<br>- 2 x 3 dagen bijhouden beweegdagboek.<br>- 2 x 7 dagen dragen van een PAM.<br><br>**Kinderen**<br>- 2 x meten van lengte, gewicht en lichaamssamenstelling (15 minuten per keer op voorschool-locatie, geen reistijd).<br><br>**Ouders/verzorgers**<br>- 2 x 3 dagen bijhouden van een eetdagboek voor hun kind(eren).<br>- 2 x 3 dagen bijhouden beweegdagboek voor hun kind(eren).<br>- 2 x beantwoorden van vragen gericht op kennis, attitude en vaardigheden t.a.v. een gezonde leefstijl (30 minuten per keer).

	- 1 x beantwoorden van vragen van de voedselfrequentievragenlijst (30 minuten).
**Risico voor de proefpersoon**	De risico's van het onderzoek zijn laag. De metingen zijn niet invasief en niet schadelijk voor de deelnemers.
**Voordelen deelname aan het onderzoek**	Met deelname aan PreSchool@HealthyWeight draagt een PMer bij aan een maatschappelijk relevant en wetenschappelijk onderzoek. Het onderzoek betreft een belangrijk onderwerp voor de professionalisering van de beroepsgroep. PMers werkzaam op voorscholen krijgen in de interventiegroep training in het ondersteunen van kinderen en hun ouders/verzorgers bij een gezonde leefstijl. PMers in de interventie- en controlegroep krijgen door het bijhouden van het eet- en beweegdagboek inzicht in hun voedings- en beweeggedrag. Daarnaast wordt indien gewenst de lichaamssamenstelling teruggekoppeld.<br>Ouders/verzorgers kunnen met deelname aan PreSchool@HealthyWeight bijdragen aan een maatschappelijk relevant en wetenschappelijk onderzoek. Door het invullen van het eet- en beweegdagboek krijgen de ouders/verzorgers inzicht in het voedings- en beweeggedrag van hun kind(eren). In de interventiegroep krijgen ouders/verzorgers tevens feedback op het voedings- en beweeggedrag van hun kind(eren).
**Nadelen deelname aan het onderzoek**	Een nadeel van PreSchool@HealthyWeight is dat het tijd kost.
**Vergoeding voor proefpersoon**	Er is geen vergoeding voor proefpersonen voor deelname aan het onderzoek.
**Administratieve aspecten**	Alle geïncludeerde voorschool-locaties krijgen een randomisatienummer. Daarnaast wordt aan alle PMers, kinderen en ouders/verzorgers een deelnemernummer toegekend. Vragenlijsten en eet- en beweegdagboeken worden gecodeerd. De papieren documenten worden bewaard in een afgesloten ruimte op de HvA, los van de getekende Informed Consent formulieren. Digitale gegevens worden bewaard in mappen op de interne schijf van de HvA. Alleen onderzoekers van PreSchool@HealthyWeight hebben toegang tot de mappen. Digitale gecodeerde gegevens worden tevens opgeslagen via SURFdrive: een veilige persoonlijke cloudopslagdienst voor het Nederlandse onderwijs en onderzoek. Gecodeerde gegevens die worden opgeslagen op SURFdrive zijn niet herleidbaar tot een individueel persoon. Alle documenten worden tot 10 jaar na het onderzoek opgeslagen.

**Publicatiebeleid en amendementen**	Publicaties in professionele- en wetenschappelijke tijdschriften worden beoogd. Daarnaast wordt nagestreefd om op lokale, landelijke en internationale podia presentaties te geven.
**Referenties**	- Alkon, A., Crowley A.A., Neelon S.E.B., Hill S., Pan Y., Nguyen V., Rose R., Savage E., Forestieri N., Shipman L., Kotch J.B. (2014). Nutrition and physical activity randomized control trial in child care centers improves knowledge, policies, and children's body mass index. BMC Public Health, 14:215.<br>- Bouthoorn, S. H., Wijtzes, A. I., Jaddoe, V.W., Hofman, A., Raat, H., & van Lenthe, F. J. (2014). Development of socioeconomic inequalities in obesity among Dutch pre-school and school-aged children. Obesity (Silver Spring, Md), 22(10), 2230-2237.<br>- Centraal Bureau voor de Statistiek. (2016). Leefstijl; overgewicht (jongeren 2 tot 25 jaar). Geraadpleegd op 25 mei 2016, van http://jeugdstatline.cbs.nl/Jeugdmonitor/publication/?DM=SLNL&PA=71851ned&D1=2&D2=0,3-5&D3=0,5-l&HDR=T,G2&STB=G1&CHARTTYPE=1&VW=T<br>- de Kroon, M. L., Renders, C. M., van Wouwe, J.P., van Buuren, S., & Hirasing, R. A. (2010). The Terneuzen Birth Cohort: BMI changes between 2 and 6 years correlate strongest with adult overweight. PloS One, 5(2), e9155.<br>- de Munter, J. S. L., van Valkengoed, I. G. M., Agyemang, C., Kunst, A. E., & Stronks, K. (2010). Large ethnic variations in recommended physical activity according to activity domains in amsterdam, the netherlands. The international Journal of Behavioral Nutrition and Physical Activity, 7, 85-5868-7-85.<br>- Dutman, A.E., Stafleu, A., Kruizinga, A., Brants, H.A.M., Westerterp, K.R., Kistemaker, C., Meuling, W.J.A., & Goldbohm, R.A. (2011). Validation of an FFQ and options for data processing using the doubly labelled water method in children. Public Health Nutrition, 14(3), 410-417.<br>- Gemeente Amsterdam. (2015). Amsterdamse Aanpak Gezond Gewicht Programmaplan 2015 – 2018. Geraadpleegd op 25 mei 2016, van https://www.amsterdam.nl/gemeente/organisatie/sociaal/onderwijs-jeugd-zorg/amsterdamse-aanpak/programma/

	- Gubbels, J. S., Kremers, S. P., Stafleu, A., Dagnelie, P. C., de Vries, N. K., & Thijs, C. (2010). Child-care environment and dietary intake of 2- and 3-year-old children. Journal of Human Nutrition and Dietetics : The Official Journal of the British Dietetic Association, 23(1), 97-101.<br>- Gubbels, J.S., Sleddens, E.F.C., Raaijmakers, L.C.H., Gies, J.M. & Kremers, S.P.J. (2015). The Child-care Food and Activity Practiced Questionnaire (CFAPQ): development and first validation steps. Public Health Nutrition, Dec 4, 1-12.<br>- Janssen, M., Toussaint, H. M., Van Willem, M., & Verhagen, E. A. (2011). PLAYgrounds: Effect of a PE playground program in primary schools on PA levels during recess in 6 to 12 year old children. Design of a prospective controlled trial. BMC Public Health, 11, 282-2458-11-282.<br>- Janssen, M., Twisk, J.W., Toussaint, H. M., van Mechelen, W., & Verhagen, E. A. (2015). Effectiveness of the PLAYgrounds programme on PA levels during recess in 6-year-old to 12-year-old children. British Journal of Sports Medicine, 49(4), 259-264.<br>- McKenzie, T.L., Marshall, S.J. Sallis, J.F., & Conway, T.L. (2000). Leisure-time physical activity in school environments: an observational study using SOPLAY. Preventive Medicine, 30(1), 70-77.<br>- Musher-Eizenman, D., Holub, S. (2007). Comprehensive Feeding Practices Questionnaire: Validation of a New Measure of Parental Feeding Practices. Journal of Pediatric Psychology, 32(8), 960-72.<br>- Nicolaou, M., Nierkens, V., & Middelkoop, B. J. C. (2013). Cultural diversity in diet and obesity [Culturele diversiteit in voeding en overgewicht]. Nederlands Tijdschrift Voor Geneeskunde, 157(18), A5807.<br>- O'Connor, T.M., Cerin, E., Hughes, S.O., Robles, J., Thompson, D.I., Mendoza, J.A., Baranowski, T., Lee, R.E. 2014. Psychometrics of the preschooler physical activity parenting practices instrument among a latino sample. The International Journal of Behavioral Nutrition and physical Activity, Jan 15, 11:3.<br>- Sarphati Amsterdam (2016). Sarphati Cohort. Geraadpleegd op 30 juni 2016, van https://www.sarphati.amsterdam/#sarphati-cohort.

	- Schönbeck, Y., Talma, H., van Dommelen, P., Bakker, B., Buitendijk, S.E., Hirasing, R.A., & van Buuren, S. (2011). Increase in prevalence of overweight in Dutch children and adolescents: a comparison of nationwide growth studies in 1980, 1997 and 2009. PLoS One, 6(11), e27608.<br>- Schönbeck, Y., van Buuren, S. (2010). Factsheet Resultaten Vijfde Landelijke Groeistudie. Geraadpleegd op 25 mei 2016, van https://www.tno.nl/media/1996/20100608-factsheet-resultaten-vijfde-landelijke-groeistudie1.pdf<br>- Schulz, J., Henderson, S.E., Sugden, D.A., & Barnett, A.L. 2011. Structural validity of the movement ABC-2 test: factor structure comparison across three age groups. Research in Developmental Disabilities, 32(4), 1361-1369.<br>- Volksgezondheidenzorg.info. (2016). Gezondheidsgevolgen bij kinderen. Geraadpleegd op 25 mei 2016, van https://www.volksgezondheidenzorg.info/onderwerp/overgewicht/cijfers-context/gezondheidsgevolgen#!node-gezondheidsgevolgen-bij-kinderen
