# Supplementary material for: The effects of a preschool-based intervention for Early Childhood Education and Care teachers in promoting healthy eating and physical activity in young children: A cluster randomised controlled trial
Source: PLoS One. 2021 Jul 23;16(7):e0255023. doi: 10.1371/journal.pone.0255023 (PMC8302250; doi:10.1371/journal.pone.0255023)
Supplement: S3 File — (PDF) [file pone.0255023.s003.pdf]

Onderzoeksprotocol / Study Protocol for the Medical Ethics Review Committee

Algemene gegevens / General information

**Titel/ Title**	PreSchool@HealthyWeight; Op weg naar een gezonde kinderopvang voor elke peuter / PreSchool@HealthyWeight; Towards a healthy child care environment for every toddler
**Datum / Date**	01-07-2016
**Versienummer / Version**	1
**Indiener / Submitter**	Dr. Ir. Peter J.M. Weijs<br>Coördinator onderzoek / Sectie Voeding en Diëtetiek / Research coordinator / Section Nutrition and Dietetics<br>Afdeling interne geneeskunde / Internal medicine<br>VUmc Amsterdam<br>De Boelelaan 1117, ZH4A12, 1081 HV Amsterdam<br>p.weijs@vumc.nl<br><br>Lector Gewichtsmanagement / Head research group Weight management<br>Hogeschool van Amsterdam / Amsterdam University of Applied Sciences<br>Faculteit Bewegen, Sport en Voeding / Faculty of Sports and Nutrition<br>Kenniscentrum Bewegen, Sport en Voeding / Knowledge centre Sports and Nutrition<br>Dr. Meurerlaan 8, 1067 SM Amsterdam<br>p.j.m.weijs@hva.nl
**Coördinerend onderzoeker / Coordinating investigator**	Dr. Ir. Martinette Streppel<br>Senior onderzoeker / Senior researcher<br>Hogeschool van Amsterdam / Amsterdam University of Applied Sciences<br>Faculteit Bewegen, Sport en Voeding / Faculty of Sports and Nutrition<br>Dr. Meurerlaan 8, 1067 SM Amsterdam<br>m.t.streppel@hva.nl
**Hoofdonderzoeker(s) / Principal investigator**	Dr. Ir. Peter J.M. Weijs
**Opdrachtgever (verrichter) / Sponsor**	Hogeschool van Amsterdam (HvA) / Amsterdam University of Applied Sciences (AUAS)<br>Hogeschool van Amsterdam

Onderzoekgegevens / Information about the study

**Rationale / Rationale**	De prevalentie van overgewicht en obesitas bij kinderen in Nederland is hoog (Schönbeck *et al*., 2011). / The prevalence of overweight and obesity among children in the Netherlands is high. Het Centraal Bureau voor de Statistiek laat op basis van zelfgerapporteerde waarden zien dat in 2015 bij 12,2% van de kinderen tussen de 2 en 9 jaar sprake was van overgewicht (Centraal Bureau voor de Statistiek, 2016). / Based on self-reported data, Statistics Netherlands shows that 12.2% of children between 2 and 9 years old were overweight in 2015. Zowel in de kinderjaren als op latere leeftijd vergroten overgewicht en obesitas de kans op gezondheidsproblemen (Volkgezondheidenzorg.info, 2016). / Both, in childhood and later in life, overweight and obesity increase the risk of having health problems.<br>Het is bekend dat (ernstig) overgewicht samenhangt met dagelijkse keuzes in voedings- en beweeggedrag en dat deze gedragingen verschillen tussen etnische en sociaaleconomische groepen (de Munter, van Valkengoed, Agyemang, Kunst, & Stronks, 2010; Nicolaou, Nierkens, & Middelkoop, 2013). / Excess weight gain is associated with daily choices in eating and physical activity behaviours and these behaviours differ between ethnic and socio-economic groups. Al op zeer jonge leeftijd zijn verschillen in Body Mass Index (BMI) tussen sociaaleconomische groepen aanwezig (Bouthoorn *et al*., 2014). / Differences in Body Mass Index (BMI) between socio-economic groups are present at an early age. Resultaten van de Vijfde Landelijke Groeistudie laten zien dat het percentage kinderen met overgewicht en obesitas hoger is bij kinderen met een Turkse of Marokkaanse afkomst dan bij Nederlandse kinderen (Schönbeck, van Buuren, 2010). / Results of the Fifth National Growth Study show that the percentage of overweight and obese children is higher among children with Turkish or Moroccan backgrounds compared to Dutch children.<br>Het leeftijdsinterval van 2 tot 6 jaar is belangrijk voor de ontwikkeling van overgewicht bij volwassenen (de Kroon, Renders, van Wouwe, van Buuren, & Hirasing, 2010). / The age range of 2 to 6 years is important for the development of adult overweight. Bovendien speelt de omgeving waarin een kind opgroeit een relevante rol bij het ontstaan van (ernstig) overgewicht. / Furthermore, the environment in which a child grows up plays a relevant role in the development of excess weight gain. Naast de thuissituatie is de kinderopvang een invloedrijke omgevingsfactor

	(Gubbels *et al*., 2010). / Next to the home environment, the child care environment is important.<br>Op voorscholen werken Pedagogisch Medewerkers (PMers) met kinderen tussen de 2,5 en 4 jaar. / At preschools, Early Childhood Education and Care (ECEC) teachers support children between 2.5 to 4 years old. PMers zijn getraind in het ondersteunen van de ontwikkeling en opvoeding van kinderen. / ECEC teachers are trained to support the development and education of children. De medewerkers kunnen invloed hebben op wat kinderen eten en drinken en hoeveel ze bewegen in de voorschoolse periode. / The teachers can affect what children eat and drink and how much the children exercise during the preschool period. Echter, PMers zijn niet opgeleid om begeleiding te geven op het gebied van leefstijl. / However, ECEC teachers are not trained to support in pursuing a healthy lifestyle. Er is sprake van handelingsverlegenheid voortkomend uit een gebrek aan kennis en aan interactievaardigheden met ouders/verzorgers omtrent dit onderwerp. / The inability to provide adequate support results from a lack of knowledge and interaction skills to communicate with parents/caregivers about this topic.<br>In opdracht van het Ministerie van Volksgezondheid, Welzijn en Sport is door het Voedingscentrum, Nederlands Jeugdinstituut, Nederlands Instituut voor Sport en Bewegen, TNO, VeiligheidNL en Pharos, vanuit het maatschappelijke belang om kinderen vanaf heel jonge leeftijd zo gezond mogelijk te laten opgroeien, een training voor PMers ontwikkeld: Een Gezonde Start 2.0 (EGS2). / Commissioned by the Ministry of Health, Welfare and Sport, The Netherlands Nutrition Centre, Netherlands Youth Institute, Netherlands Institute for Sport and Physical Activity, TNO, VeiligheidNL and Pharos developed, based on the social interest of allowing children to grow up as healthy as possible from a very young age, a training for ECEC teachers: A Healthy Start 2.0 (AHS). EGS2 leert PMers hoe zij samen met ouders/verzorgers een gezonde, actieve en veilige omgeving kunnen bieden aan kinderen en hoe zij zelf het gezonde voorbeeld kunnen geven. / AHS will learn ECEC teachers how they can, together with parents, provide a healthy, active and safe environment for children and how to set a healthy example. Het landelijke programma is primair gericht op interactievaardigheden met ouders/verzorgers. / The national program focuses on interaction skills to communicate with parents/caregivers. Met een aangepaste versie van de aangetoond effectieve PLAYgrounds interventie wordt, door een uitnodigende en

	gestructureerde inrichting van de buitenruimte en stimulans van het actief gebruik van de buitenruimte, lichamelijke beweging van kinderen gestimuleerd (Janssen, Toussaint, van Willem, & Verhagen, 2011; Janssen, Twisk, Toussaint, van Mechelen, & Verhagen, 2015). / With a modified version of the effective PLAYgrounds intervention, the physical activity of children will be stimulated through an inviting structure and active use of the playground. Middels instructie van een trainer krijgt een PMer handvatten aangereikt om aan de slag te gaan met het beweeggedrag van kinderen in de buitenruimte. / Via instructions of a trainer, ECEC teachers will get tools to stimulate physical activity on the playground. PreSchool@HealthyWeight onderzoekt wat de PMer in de voorschool nodig heeft aan kennis, attitude en vaardigheden om een goede ondersteuning te kunnen geven aan kinderen en hun ouders/verzorgers bij een gezonde leefstijl. / PreSchool@HealthyWeight examines what ECEC teachers need in terms of knowledge, attitude and practices in order to be able to provide support to children and their parents/caregivers in pursuing a healthy lifestyle. Met de bestaande training EGS2 en een aangepaste versie van de aangetoond effectieve PLAYgrounds training beoogt het onderzoek het vertrouwen van PMers in het geven van goede ondersteuning aan kinderen en hun ouders/verzorgers bij een gezonde leefstijl te vergroten. / With the existing training AHS and modified version of the effective PLAYgrounds intervention, the study aims to increase the level of confidence of ECEC teachers in supporting children and their parents/caregivers in pursuing a healthy lifestyle. Er wordt geambieerd een bijdrage te leveren aan het bevorderen van een gezonde (gewichts)ontwikkeling van kinderen en het terugdringen van gezondheidsverschillen tussen kinderen met diverse sociaal-economische en cultureel-etnische achtergronden. / The aim is to contribute to the promotion of a healthy (weight) development of children and minimise health inequalities in children with diverse socio-economic and cultural backgrounds.<br>Het onderzoek wordt grotendeels uitgevoerd op voorscholen in Amsterdam Nieuw-West. In Nieuw-West is het probleem van overgewicht en obesitas groter dan in andere stadsdelen, dit hangt sterk samen met het lage opleidingsniveau, de lage inkomens en etniciteit van inwoners in de wijk (Gemeente Amsterdam, 2015).

	/ The study will be mainly conducted at preschools in Amsterdam Nieuw-West. In Nieuw-West the problem of overweight and obesity is bigger compared to other city districts. This is associated to the low socio-economic status and ethnicity of families in this district.
**Doel / Objective**	Het primaire doel van de studie is het vergroten van het vertrouwen van PMers, werkzaam bij voorscholen van kinderopvangorganisatie Impuls in Amsterdam Nieuw-West, in het goed kunnen ondersteunen van kinderen en hun ouders/verzorgers bij een gezonde leefstijl.
	De secundaire doelstelling is het meten van het effect van het trainen van PMers in het geven van een goede ondersteuning t.a.v. een gezonde leefstijl op:<br>- kennis, attitude en vaardigheden van PMers en ouders/verzorgers t.a.v. een gezonde leefstijl;<br>- lichaamssamenstelling en BMI van PMers en kinderen;<br>- voedings- en beweeggedrag van PMers en kinderen.<br>/ The primary objective of this study is to increase the level of confidence of Early Childhood Education and Care (ECEC) teachers in supporting children and their parents/caregivers in pursuing a healthy lifestyle. The secondary objective is to gain insight in the effect of training ECEC teachers in supporting children and their parents/caregivers regarding a healthy life style on:<br>- knowledge, attitude and practices of ECEC teachers and parents/ caregivers concerning a healthy lifestyle;<br>- body composition and BMI of ECEC teachers and children;<br>- eating behaviour and physical activity level of ECEC teachers and children.
**Studie design / Study design**	Het onderzoek betreft een cluster gerandomiseerde interventie studie. Voor de studie worden voorschool-locaties van kinderopvangorganisatie Impuls willekeurig toegewezen aan de interventie- of controlegroep. / Cluster randomised controlled trial. For this study preschools of child care organisation Impuls will be randomly allocated to an intervention or control group.
**Studie populatie / Study population**	De studiepopulatie bestaat uit PMers, kinderen en hun ouders/verzorgers van voorscholen van kinderopvangorganisatie Impuls in Amsterdam Nieuw-West. / ECEC teachers, children and their parents/caregivers at preschools of child care organisation Impuls in Amsterdam Nieuw-West.

**Inclusie criteria / Inclusion criteria**	- PMers met een vast dienstverband bij kinderopvangorganisatie Impuls in Amsterdam Nieuw-West.<br>- PMers met een leeftijd ≥ 18 jaar.<br>- PMers die ≥ de helft van hun dienstverband werken bij een voorschool-locatie van kinderopvangorganisatie Impuls in Amsterdam Nieuw-West.<br>- Kinderen met een leeftijd ≥ 2,5 jaar en ≤ 3 jaar.<br>- Kinderen en hun ouders/verzorgers die begeleiding krijgen van een geïncludeerde PMer op een voorschool-locatie van kinderopvangorganisatie Impuls in Amsterdam Nieuw-West.<br>/<br>- ECEC teachers with a permanent contract at child care organisation Impuls located in Amsterdam Nieuw-West.<br>- ECEC teachers > 18 years.<br>- ECEC teachers who work > half of their employment at a preschool of child care organisation Impuls located in Amsterdam Nieuw-West.<br>- Children between the age of 2.5 to 3 years.<br>- Children and their parents/caregivers who are under the care of included ECEC teachers of child care organisation Impuls located in Amsterdam Nieuw-West.
**Exclusie criteria / Exclusion criteria**	- PMers werkzaam bij meerdere voorschool-locaties (zowel een interventie- als een controlelocatie) van kinderopvangorganisatie Impuls.<br>- Voorschool-locaties waar al interventies plaatsvinden met betrekking tot een gezonde leefstijl waarvan de onderzoekers verwachten dat ze interveniëren met EGS2 en PLAYgrounds.<br>/<br>- ECEC teachers who work at multiple preschool locations (on intervention and control locations) of child care organisation Impuls.<br>- Preschool locations where other lifestyle interventions take place that may intervene with AHS and PLAYgrounds.
**Aantal proefpersonen/ sample grootte / Sample size**	Voor het onderzoek worden voorschool-locaties van kinderopvangorganisatie Impuls willekeurig toegewezen aan de interventie- of controlegroep. / For the study, preschools of child care organisation Impuls will be randomly allocated to the intervention or control group. Om een antwoord te geven op de hoofdvraag van het onderzoek worden 120 PMers geïncludeerd (60 PMers in de interventiegroep en 60 PMers in de controlegroep). / To answer the main

	research question, 120 ECEC teachers will be included (60 ECEC teachers in the intervention group and 60 ECEC teachers in the control group). De sample grootte is gebaseerd op een medium effect grootte (Cohen's d: 0.50), een 1-zijdige alpha van 5%, en een power van 80%. Daarnaast is er gecorrigeerd voor
	het design effect (gemiddelde cluster grootte: 3, ICC: 0.05) en rekening gehouden met 10% uitval. / The sample size is based on a medium effect size (Cohen's d: 0.50), a 1-sided alpha of 5% and a power of 80%, taken into account a design effect (mean cluster size = 3, ICC: 0.05) and a 10% drop-out rate. Om antwoord te geven op de secundaire onderzoeksvragen worden per PMer 2 kinderen en hun ouders/verzorgers geïncludeerd. In totaal gaat het om 240 kinderen en hun ouders/verzorgers (120 kinderen en hun ouders/verzorgers in de interventiegroep en 120 kinderen en hun ouders/verzorgers in de controlegroep). / To answer the secondary research questions, per ECEC teacher, 2 children and their parents/caregivers will be included. In total, it concerns 240 children and their parents/caregivers (120 children and their parents/caregivers in the intervention group and 120 children and their parents/caregivers in the control group). Met 120 kinderen per groep is het mogelijk om, met een 2-zijdige alpha van 5% en een power van 80%, een klein tot medium effect grootte (Cohen's d: 0.35-0.40) aan te tonen. / With 120 children per group, it is possible to show, with a 2-sided alpha of 5% and a power of 80%, a small to medium effect size (Cohen's d: 0.35 - 0.40). Dit beoogde aantal kinderen is vergelijkbaar met de sample grootte van een gecombineerde voedings- en beweeginterventie waar na 7 maanden follow-up een significant verschil in BMI z-score werd aangetoond tussen de interventie- en controlegroep (Alkon *et al.*, 2013). / This sample size is comparable to the sample size of a combined nutrition and physical activity intervention where a significant difference in BMI z-score was found between the intervention and control group after 7 months of follow-up.

**Werving proefpersonen / Recruitment**	Alle geschikte PMers van een geïncludeerde voorschool-locatie worden via een wijkmanager en/of coach van kinderopvangorganisatie Impuls (leidinggevende) mondeling ingelicht over het onderzoek. Daarnaast ontvangen PMers een informatiebrief met uitgebreide uitleg betreffende PreSchool@HealthyWeight. / All ECEC teachers at included preschools will be informed about the study via their district manager or coach of child care organisation Impuls. Furthermore, the ECEC teachers will receive an information letter with a detailed explanation about the study. Vervolgens bezoeken onderzoekers van de HvA voorschool-locaties om mondeling toelichting te geven en eventuele vragen te beantwoorden. / Next, AUAS researchers will visit the preschools to provide oral explanation and answer questions. Voorafgaand aan de metingen wordt schriftelijk informed consent verkregen. / Written informed consent will be obtained.<br>Ouders/verzorgers worden via PMers mondeling op de hoogte gebracht van het onderzoek. / Parents/caregivers will be informed about the study via ECEC teachers. Daarnaast zal er via een nieuwsbrief van Impuls informatie worden verstrekt aan ouders/verzorgers. / Furthermore, parents/caregivers will receive information via a newsletter. Bij de voorscholen van kinderopvangorganisatie Impuls bestaat de afspraak dat ouders/verzorgers van de kinderen minimaal 15 minuten aanwezig zijn op de voorschool. / At the preschools of child care organisation Impuls, it is agreed that parents/caregivers are present at the preschool for at least 15 minutes. In deze 15 minuten zal door onderzoekers van de HvA mondeling en schriftelijk (via een brief) uitgebreide informatie over het onderzoek worden gegeven. / In this 15 minutes, AUAS researchers will provide oral and written information (via a letter) about the study. Bij een volgend bezoek kunnen eventuele vragen worden beantwoord en wordt schriftelijk informed consent verkregen. / In a next visit, questions will be answered and written informed consent will be obtained.
**Interventie / Intervention**	PreSchool@HealthyWeight betreft onderzoek bij 3 doelgroepen: PMers, kinderen en hun ouders/verzorgers. / PreSchool@HealthyWeight concerns research at 3 populations: ECEC teachers, children and their parents/caregivers. Voor PMers bestaat het onderzoek uit een nulmeting, tussenmeting en nameting. / For ECEC teachers the study consist of a baseline measurement, follow-up measurement and close out measurement. Bij kinderen en hun ouders/verzorgers wordt alleen een nul- en nameting uitgevoerd. / For children and their parents/caregivers only

	baseline and close out measurements will be carried out. Na de nulmeting bij PMers, kinderen en hun ouders/verzorgers, starten PMers op een interventielocatie met het programma EGS2. / After baseline measurements, ECEC teachers at intervention preschools will start with the AHS training. De training bestaat uit drie bijeenkomsten en gaat aan de hand van theorie en opdrachten in op een gezonde leefstijl van PMers, kinderen en hun ouders/verzorgers. / The training consists of three meetings and based on theory and assignments, ECEC teachers will learn about pursuing a healthy lifestyle. Bijeenkomst 1 is gericht op een gezonde leefstijl van kinderen en PMers zelf. / Meeting 1 focuses on a healthy lifestyle for children and on the ECEC teachers' personal lifestyle. In bijeenkomst 2 wordt aandacht besteed aan de interactie met kinderen rondom een gezonde leefstijl. / In meeting 2, the interaction with children regarding a healthy lifestyle will be discussed. De laatste bijeenkomst gaat over de interactie met ouders/verzorgers t.a.v. een gezonde leefstijl van hun kind(eren). / The third meeting will concern the interaction with parents/caregivers regarding a healthy lifestyle for their child. De training begint met een instaptoets voor het toetsten van initieel kennisniveau van PMers en wordt afgesloten met een eindtoets. / The training starts with an test to examine the initial knowledge level of ECEC teachers and concludes with a final test. Aansluitend op het programma vindt een periode plaats (± 3 maanden) waarin PMers verkregen kennis en vaardigheden t.a.v. een gezonde leefstijl in de praktijk brengen. / After the training, there is a period (± 3 months) in which ECEC teachers can put the gained knowledge into practice. PMers krijgen een actieve rol door feedback te geven aan ouders/verzorgers op het voedings- en beweeggedrag van hun kind(eren). / ECEC teachers can have an active role by giving feedback to parents/caregivers on the eating behaviour and physical activity level of their child(ren). Deze feedback is gebaseerd op een uitgebreide analyse van het voedings- en beweeggedrag van de kinderen (gegevens verzameld bij de nulmeting van kinderen en hun ouders/verzorgers) en wordt uitgevoerd door onderzoekers en studenten van de HvA. / The feedback is based on an analyses of the eating and physical activity behaviours of children (data obtained at baseline and close out measurements by researchers and students of the AUAS).

	Om het effect van de training EGS2 te meten wordt een tussenmeting verricht bij PMers. / To examine the effects of the AHS training, an follow-up measurement for ECEC teachers will be carried out. Hierbij zullen de resultaten van de instap- en eindtoets van EGS2 worden meegenomen. / The results of the entry and final test will be included. Na de tussenmetingen volgt een periode (± 3 maanden) waarin een aangepaste versie van de aangetoond effectieve PLAYgrounds training wordt uitgevoerd. / After the follow-up measurement, a period (± 3 months) with the modified PLAYgrounds intervention will follow. Middels instructie door een trainer gaan de PMers aan de slag met beweeggedrag van de kinderen in de buitenruimte. / Via instructions of a trainer, ECEC teachers will learn about stimulating physical activity of children on the playground.<br>Op voorschool-locaties in de controlegroep worden de trainingen EGS2 en PLAYgrounds niet aangeboden en krijgen ouders/verzorgers geen feedback op het voedings- en beweeggedrag van hun kind(eren). / At preschools in the control group, ECEC teachers will not follow the AHS training and PLAYgrounds intervention and parents/caregivers will not receive feedback on the eating and physical activity behaviours of their child(ren). Met een nameting bij PMers, kinderen en hun ouders/verzorgers wordt de dataverzameling van het onderzoek afgesloten. / Data collection will be concluded with close out measurements for ECEC teachers, children and their parents/caregivers.<br><br>Naast PreSchool@HealthyWeight zullen de kinderen, als Sarphati-cohort, onderdeel uitmaken van het Sarphati Amsterdam (Sarphati Amsterdam, 2016). / In addition to PreSchool@HealthyWeight, the children will be part of the Sarphati Amsterdam Cohort. Hiermee is het mogelijk om effecten op langere termijn vast te stellen. / This makes it possible to determine long-term effects.

	De nul- en nameting voor PMers bestaat uit: / The baseline and close out measurements for ECEC teachers will consist of:<br>- Het invullen van een vragenlijst (Gubbels, Sleddens, Raaijmakers, Gies, & Kremers, 2015) gericht op kennis, attitude en vaardigheden t.a.v. een gezonde leefstijl. In de vragenlijst komt het vertrouwen van PMers in het goed kunnen ondersteunen van kinderen en hun ouders/verzorgers bij een gezonde leefstijl aan bod. Tevens worden bij de nulmeting in deze vragenlijst enkele demografische kenmerken nagevraagd. / Questionnaires about knowledge, attitude and practices regarding a healthy lifestyle. In the questionnaires also the level of confidence of ECEC teachers in supporting children and their parents/caregivers in pursuing a healthy lifestyle will be checked. Furthermore, demographic characteristics will be obtained at baseline.<br>- Het meten van lengte en gewicht (voor het berekenen van de BMI). / Height and weight measurements (to calculate BMI).<br>- Het meten van lichaamssamenstelling middels Bio-elektrische Impedantie Analyse (BIA). / Body composition measurements via Bioelectrical Impedance Analysis (BIA).<br>- Het invullen van een 3 daags eetdagboek. / 3-day food records.<br>- Het invullen van een 3 daags beweegdagboek. / 3-day physical activity records.<br>- Het meten van fysieke activiteit op 7 dagen met een Physical Activity Monitor (PAM). / Physical activity measurements via a Physical Activity Monitor (PAM) – 7 days.<br>Getrainde studenten van de HvA meten lengte, gewicht en lichaamssamenstelling van een PMer op de voorschool-locatie zelf. / Trained students of the AUAS will measure height, weight and body composition of the ECEC teachers at preschool. Na de metingen wordt op papier een vragenlijst en eet- en beweegdagboek met bijhorende informatie verstrekt. / After the measurements, a questionnaire, food record and physical activity record will be provided on paper. Daarnaast wordt een PAM meegegeven. / Furtermore, a PAM will be given. Na een week haalt een student de vragenlijst, de PAM, het eetdagboek en het beweegdagboek op bij de voorschool-locatie. / After a week, a student will pick up the questionnaire, PAM, food record and physical activity record at preschool. Hierbij controleert de student de ingevulde vragenlijsten op volledigheid. / The student will check if a questionnaire is completed.

	De tussenmeting voor PMers bestaat uit: / The follow-up measurements for ECEC teachers will consist of:<br>- Het invullen van een vragenlijst (Gubbels, Sleddens, Raaijmakers, Gies, & Kremers, 2015) gericht op kennis, attitude en vaardigheden t.a.v. een gezonde leefstijl . In de vragenlijst komt het vertrouwen van PMers in het goed kunnen ondersteunen van kinderen en hun ouders/verzorgers bij een gezonde leefstijl aan bod. / Questionnaires about knowledge, attitude and practices regarding a healthy lifestyle. In the questionnaires also the level of confidence of ECEC teachers in supporting children and their parents/caregivers in pursuing a healthy lifestyle will be checked.<br>- Het meten van lengte en gewicht (voor het berekenen van de BMI). / Height and weight measurements (to calculate BMI).<br>- Het meten van lichaamssamenstelling middels BIA. / Body composition measurements via Bioelectrical Impedance Analysis (BIA).<br>Getrainde studenten van de HvA meten lengte, gewicht en lichaamssamenstelling van een PMer op de voorschool-locatie zelf. / Trained students of the AUAS will measure height, weight and body composition of the ECEC teachers at preschool. Na de metingen wordt op papier een vragenlijst met bijhorende informatie verstrekt. / After the measurements, a questionnaire will be provided on paper. Na een week haalt een student de vragenlijst op bij de voorschool-locatie. / After a week, a student will pick up the questionnaires at preschool. Hierbij controleert de student de ingevulde vragenlijsten op volledigheid. / The student will check if a questionnaire is completed.<br><br>De nul- en nameting voor kinderen bestaat uit: / The baseline and close out measurements for children will consist of:<br>- Het meten van lengte en gewicht (voor het berekenen van de BMI). / Height and weight measurements (to calculate BMI).<br>- Het meten van lichaamssamenstelling middels BIA. / Body composition measurements via Bioelectrical Impedance Analysis (BIA).<br>- Een observatie van beweeggedrag (Schulz, Henderson, Sugden, & Barnett, 2011; McKenzie, Marshall, Sallis, & Conway, 2000). / Observations of physical activity.<br>De metingen en observaties vinden plaats op de voorschool-locatie zelf en worden uitgevoerd door getrainde studenten van de HvA. / The measurements and observations will take place at preschools and will be carried out by trained students of the AUAS.

	De nul- en nameting voor ouders/verzorgers bestaat uit: / The baseline and close out measurements for parents/caregivers will consist of:<br>- Het invullen van een 3 daags eetdagboek van de kinderen. / 3-day food records for their child.<br>- Het invullen van een 3 daags beweegdagboek van de kinderen. / 3-day physical activity records for their child.<br>- Een voedselfrequentievragenlijst (Dutman *et al*., 2011) van de kinderen (de vragenlijst wordt mondeling afgenomen door studenten van de HvA). Naast het eetdagboek (gericht op dag tot dag variatie in voedingsinname), geeft de gevalideerde voedselfrequentievragenlijst aanvullende informatie over voedingspatronen van de kinderen. De verkregen informatie wordt gebruikt door onderzoekers en studenten van de HvA voor het opstellen van feedback aan ouders/verzorgers. De voedselfrequentievragenlijst wordt alleen bij de nulmeting afgenomen. / Food Frequency Questionnaires for their child (the questionnaire will be administered orally by AUAS students). In addition to the food record (focusing on day-to-day variation in food intake), the validated Food Frequency Questionnaires provides additional information about the children's dietary patterns. The obtained information will be used by researchers and students of the AUAS to prepare feedback for parents/caregivers. The Food Frequency Questionnaires will only be carried out at baseline measurements.<br>- Een vragenlijst (Musher-Eizenman, Holub, 2007; O'Connor *et al*., 2014) gericht op kennis, attitude, vaardigheden en ouderbetrokkenheid t.a.v. een gezonde leefstijl van het kind. Daarnaast worden bij de nulmeting in deze vragenlijst enkele demografische kenmerken nagevraagd (de vragenlijst wordt mondeling afgenomen door studenten van de HvA). / Questionnaires about knowledge, attitude and practices regarding a healthy lifestyle. At baseline, also demographic characteristics will be obtained (the questionnaire will be administered orally by AUAS students).<br>Bij kinderopvangorganisatie Impuls bestaat de afspraak dat ouders/verzorgers minimaal 15 minuten aanwezig zijn op de voorschool. / At the preschools of child care organisation Impuls, it is agreed that parents/caregivers are present at the preschool for at least 15 minutes. Deze tijd kan gebruikt worden voor het afnemen van de vragenlijsten. / This time can be used to administer the questionnaires. Tevens wordt op papier het eet- en beweegdagboek met bijhorende informatie verstrekt. /

	Furthermore, the food and physical activity records will be provided on paper. Na een week haalt een student het eetdagboek en het beweegdagboek op bij de voorschool-locatie. / After a week, a student will pick up the food and physical activity records at preschool. Hierbij controleert de student de ingevulde dagboeken op volledigheid. / The student will check if the records are completed.

**Studie eindpunten / Study endpoints**	Het vertrouwen van PMers in het goed kunnen ondersteunen van kinderen en hun ouders/verzorgers bij een gezonde leefstijl op een 10-puntsschaal. / The level of confidence (10 point Likert scale) of ECEC teachers in supporting children and their parents/caregivers to pursue a healthy lifestyle.
**Studie parameters / Study parameters**	De volgende secundaire variabelen worden meegenomen in PreSchool@HealthyWeight:<br><br>**PMers / ECEC teachers**<br>- BMI (kg/m^2)<br>- Vetmassa (kg) / fat mass (kg)<br>- Vetvrijemassa (kg) / fat free mass (kg)<br>- Energie (kcal) / energy intake (kcal)<br>- Eiwit (g) / protein intake (g)<br>- Vet (g) / fat intake (g)<br>- Verzadigd vet (g) / saturated fat intake (g)<br>- Enkelvoudig onverzadigd vet (g) / monounsaturated fat intake (g)<br>- Meervoudig onverzadigd vet (g) / polyunsaturated fat intake (g)<br>- Transvet (g) / trans fat intake (g)<br>- Koolhydraten (g) / carbohydrates intake (g)<br>- Vezels (g) / fiber intake (g)<br>- Physical Activity Level / Physical Activity Level<br>- Nederlandse Norm Gezond Bewegen / Dutch recommendations for physical activity levels<br>- Kennis, attitude en vaardigheden t.a.v. een gezonde leefstijl / knowledge, attitude and practices regarding a healthy lifestyle<br><br>**Kinderen / Children**<br>- BMI (kg/m^2)<br>- Vetmassa (kg) / fat mass (kg)<br>- Vetvrijemassa (kg) / fat free mass (kg)<br>- Energie (kcal) / energy intake (kcal)<br>- Eiwit (g) / protein intake (g)<br>- Vet (g) / fat intake (g)<br>- Verzadigd vet (g) / saturated fat intake (g)<br>- Enkelvoudig onverzadigd vet (g) / monounsaturated fat intake (g)<br>- Meervoudig onverzadigd vet (g) / polyunsaturated fat intake (g)<br>- Transvet (g) / trans fat intake (g)<br>- Koolhydraten (g) / carbohydrates intake (g)<br>- Vezels (g) / fiber intake (g)<br>- Voedingspatronen / food patterns

	- Physical Activity Level/ Physical Activity Level<br>- Nederlandse Norm Gezond Bewegen Dutch recommendations for physical activity levels<br>- Motorische ontwikkeling / motor development<br><br>**Ouders/verzorgers / Parents/caregivers**<br>- Kennis, attitude, vaardigheden en ouderbetrokkenheid t.a.v. een gezonde leefstijl / knowledge, attitude, practices and parental engagement regarding a healthy lifestyle
**Statistische analyses / Statistical analysis**	De statistische analyses worden uitgevoerd met de software IBM SPSS Statistics 22.0. / Statistical analysis will be performed with IBM Statistics 22.0. Baseline-karakteristieken worden weergegeven met beschrijvende statistiek. / Baseline characteristics will be presented using descriptive statistics. Een ongepaarde t-toets of Mann-Whitney U toets wordt gebruikt om de baseline-karakteristieken van continue variabelen tussen de interventie- en controle groep te vergelijken. / An independent t-test or Mann-Whitney U test will be used to compare the baseline characteristics of the intervention and control group. Voor categorische variabelen wordt de Chi-kwadraat toets of een Fisher's exact toets uitgevoerd. / For categorical variables, the Chi-square or Fisher's exact test will be used.<br>Veranderingen over tijd binnen en tussen de groepen worden geanalyseerd met multi-level analyse, waarbij rekening wordt gehouden met de afhankelijkheid van de metingen binnen een voorschool-locatie. / Changes over time within and between groups will be analysed with multi-level analysis, taken into account the dependence of the measurements within preschools.

**Belasting voor de proefpersoon / Burden for subjects**	**PMers / ECEC teachers**<br>- 3 x invullen van een vragenlijst gericht op kennis, attitude en vaardigheden t.a.v. een gezonde leefstijl (30 minuten per keer). / 3 x questionnaire about knowledge, attitude and practices regarding a healthy lifestyle (30 minutes per questionnaire).<br>- 3 x meten van lengte, gewicht en lichaamssamenstelling (15 minuten per keer op voorschool-locatie, geen reistijd). / 3 x height, weight, body composition measurements (15 minutes per moment at preschool, no travel time).<br>- 2 x 3 dagen bijhouden van een eetdagboek. / 2 x 3-day food record.<br>- 2 x 3 dagen bijhouden beweegdagboek. / 2 x 3-day physical activity record.<br>- 2 x 7 dagen dragen van een PAM. / 2 x PAM for 7 days.<br><br>**Kinderen / Children**<br>- 2 x meten van lengte, gewicht en lichaamssamenstelling (15 minuten per keer op voorschool-locatie, geen reistijd). / 2 x height, weight, body composition measurements (15 minutes per moment at preschool, no travel time).<br><br>**Ouders/verzorgers / Parents/caregivers**<br>- 2 x 3 dagen bijhouden van een eetdagboek voor hun kind(eren). / 2 x 3 day food record for their child(ren).<br>- 2 x 3 dagen bijhouden beweegdagboek voor hun kind(eren). / 2 x 3 day physical activity record for their child(ren).<br>- 2 x beantwoorden van vragen gericht op kennis, attitude en vaardigheden t.a.v. een gezonde leefstijl (30 minuten per keer). / 2 x questionnaire about knowledge, attitude and practices regarding a healthy lifestyle (30 minutes per questionnaire).
	- 1 x beantwoorden van vragen van de voedselfrequentievragenlijst (30 minuten). / 1 x Food Frequency Questionnaire (30 minutes).
**Risico voor de proefpersoon / Risk for subjects**	De risico's van het onderzoek zijn laag. De metingen zijn niet invasief en niet schadelijk voor de deelnemers. / The risks of the study are low. The measurements are non-invasive and not harmful to the subjects.

**Voordelen deelname aan het onderzoek/ Advantages of participating in the study**	Met deelname aan PreSchool@HealthyWeight draagt een PMer bij aan een maatschappelijk relevant en wetenschappelijk onderzoek. / By participating in PreSchool@HealthyWeight, an ECEC teacher contributes to a socially relevant and scientific study. Het onderzoek betreft een belangrijk onderwerp voor de professionalisering van de beroepsgroep. / The study concerns an important topic for the professionalisation of the ECEC profession. PMers werkzaam op voorscholen krijgen in de interventiegroep training in het ondersteunen van kinderen en hun ouders/verzorgers bij een gezonde leefstijl. / ECEC teachers at preschools in the intervention group will be provided with training to support children and their parents/caregivers in pursuing a healthy lifestyle. PMers in de interventie- en controlegroep krijgen door het bijhouden van het eet- en beweegdagboek inzicht in hun voedings- en beweeggedrag. / ECEC teachers in the intervention and control group will gain insight in their eating and physical activity behaviours by filling in a food/physical activity record. Daarnaast wordt indien gewenst de lichaamssamenstelling teruggekoppeld. / Furthermore, it is possible to get feedback on their body composition.<br>Ouders/verzorgers kunnen met deelname aan PreSchool@HealthyWeight bijdragen aan een maatschappelijk relevant en wetenschappelijk onderzoek. By participating in PreSchool@HealthyWeight, parents/caregivers can contribute to a socially relevant and scientific study Door het invullen van het eet- en beweegdagboek krijgen de ouders/verzorgers inzicht in het voedings- en beweeggedrag van hun kind(eren). / Parents/caregivers will gain insight in the eating and physical activity behaviours of their child by filling in a food/physical activity record. In de interventiegroep krijgen ouders/verzorgers tevens feedback op het voedings- en beweeggedrag van hun kind(eren). / In the intervention group, parents/caregivers will receive feedback on the eating and physical activity behaviours of their child.
**Nadelen deelname aan het Onderzoek / Disadvantages of participating in the study**	Een nadeel van PreSchool@HealthyWeight is dat het tijd kost. / A disadvantage of PreSchool@HealthyWeight is that it takes time.
**Vergoeding voor proefpersoon / Compensation for subjects**	Er is geen vergoeding voor proefpersonen voor deelname aan het onderzoek. / There is no compensation for participation in the study.

**Administratieve aspecten / Administrative aspects**	Alle geïncludeerde voorschool-locaties krijgen een randomisatienummer. / All included preschools will receive a randomisation number. Daarnaast wordt aan alle PMers, kinderen en ouders/verzorgers een deelnemernummer toegekend. Furthermore, a participant number will be assigned to all ECEC teachers, children and parents/caregivers. Vragenlijsten en eet- en beweegdagboeken worden gecodeerd. / Questionnaires and food/physical activity records will be coded. De papieren documenten worden bewaard in een afgesloten ruimte op de HvA, los van de getekende Informed Consent formulieren. / The data on paper will be stored in a locked room at the AUAS, separate from signed Informed Consent forms. Digitale gegevens worden bewaard in mappen op de interne schijf van de HvA. / Digital data will be stored in folders on the internal drive of the AUAS. Alleen onderzoekers van PreSchool@HealthyWeight hebben toegang tot de mappen. / Only the researchers of PreSchool@HealthyWeight will have access to the folders. Digitale gecodeerde gegevens worden tevens opgeslagen via SURFdrive: een veilige persoonlijke cloudopslagdienst voor het Nederlandse onderwijs en onderzoek. / Coded digital data will also be stored on SURFdrive: a secure personal cloud storage service for Dutch education and research. Gecodeerde gegevens die worden opgeslagen op SURFdrive zijn niet herleidbaar tot een individueel persoon. / Coded data on SURFdrive will not be traceable to an individual subject. Alle documenten worden tot 10 jaar na het onderzoek opgeslagen. / All documents will be stored up to 10 years after the end of the study.

**Publicatiebeleid en amendementen / Publication Policy and Amendments**	Publicaties in professionele- en wetenschappelijke tijdschriften worden beoogd. Daarnaast wordt nagestreefd om op lokale, landelijke en internationale podia presentaties te geven. / Publications in professional and scientific journals are intended. In addition, it is aimed to present results on local, national and international conferences.
**Referenties / Reference list**	- Alkon, A., Crowley A.A., Neelon S.E.B., Hill S., Pan Y., Nguyen V., Rose R., Savage E., Forestieri N., Shipman L., Kotch J.B. (2014). Nutrition and physical activity randomized control trial in child care centers improves knowledge, policies, and children's body mass index. BMC Public Health, 14:215.<br>- Bouthoorn, S. H., Wijtzes, A. I., Jaddoe, V.W., Hofman, A., Raat, H., & van Lenthe, F. J. (2014). Development of socioeconomic inequalities in obesity among Dutch pre-school and school-aged children. Obesity (Silver Spring, Md), 22(10), 2230-2237.<br>- Centraal Bureau voor de Statistiek. (2016). Leefstijl; overgewicht (jongeren 2 tot 25 jaar). Geraadpleegd op 25 mei 2016, van http://jeugdstatline.cbs.nl/Jeugdmonitor/publication/?DM=SLNL&PA=71851ned&D1=2&D2=0,3-5&D3=0,5-l&HDR=T,G2&STB=G1&CHARTTYPE=1&VW=T<br>- de Kroon, M. L., Renders, C. M., van Wouwe, J.P., van Buuren, S., & Hirasing, R. A. (2010). The Terneuzen Birth Cohort: BMI changes between 2 and 6 years correlate strongest with adult overweight. PloS One, 5(2), e9155.<br>- de Munter, J. S. L., van Valkengoed, I. G. M., Agyemang, C., Kunst, A. E., & Stronks, K. (2010). Large ethnic variations in recommended physical activity according to activity domains in amsterdam, the netherlands. The international Journal of Behavioral Nutrition and Physical Activity, 7, 85-5868-7-85.<br>- Dutman, A.E., Stafleu, A., Kruizinga, A., Brants, H.A.M., Westerterp, K.R., Kistemaker, C., Meuling, W.J.A., & Goldbohm, R.A. (2011). Validation of an FFQ and options for data processing using the doubly labelled water method in children. Public Health Nutrition, 14(3), 410-417.<br>- Gemeente Amsterdam. (2015). Amsterdamse Aanpak Gezond Gewicht Programmaplan 2015 – 2018. Geraadpleegd op 25 mei 2016, van https://www.amsterdam.nl/gemeente/organisatie/sociaal/onderwijs-jeugd-zorg/amsterdamse-aanpak/programma/

	- Gubbels, J. S., Kremers, S. P., Stafleu, A., Dagnelie, P. C., de Vries, N. K., & Thijs, C. (2010). Child-care environment and dietary intake of 2- and 3-year-old children. Journal of Human Nutrition and Dietetics : The Official Journal of the British Dietetic Association, 23(1), 97-101.<br>- Gubbels, J.S., Sleddens, E.F.C., Raaijmakers, L.C.H., Gies, J.M. & Kremers, S.P.J. (2015). The Child-care Food and Activity Practiced Questionnaire (CFAPQ): development and first validation steps. Public Health Nutrition, Dec 4, 1-12.<br>- Janssen, M., Toussaint, H. M., Van Willem, M., & Verhagen, E. A. (2011). PLAYgrounds: Effect of a PE playground program in primary schools on PA levels during recess in 6 to 12 year old children. Design of a prospective controlled trial. BMC Public Health, 11, 282-2458-11-282.<br>- Janssen, M., Twisk, J.W., Toussaint, H. M., van Mechelen, W., & Verhagen, E. A. (2015). Effectiveness of the PLAYgrounds programme on PA levels during recess in 6-year-old to 12-year-old children. British Journal of Sports Medicine, 49(4), 259-264.<br>- McKenzie, T.L., Marshall, S.J. Sallis, J.F., & Conway, T.L. (2000). Leisure-time physical activity in school environments: an observational study using SOPLAY. Preventive Medicine, 30(1), 70-77.<br>- Musher-Eizenman, D., Holub, S. (2007). Comprehensive Feeding Practices Questionnaire: Validation of a New Measure of Parental Feeding Practices. Journal of Pediatric Psychology, 32(8), 960-72.<br>- Nicolaou, M., Nierkens, V., & Middelkoop, B. J. C. (2013). Cultural diversity in diet and obesity [Culturele diversiteit in voeding en overgewicht]. Nederlands Tijdschrift Voor Geneeskunde, 157(18), A5807.<br>- O'Connor, T.M., Cerin, E., Hughes, S.O., Robles, J., Thompson, D.I., Mendoza, J.A., Baranowski, T., Lee, R.E. 2014. Psychometrics of the preschooler physical activity parenting practices instrument among a latino sample. The International Journal of Behavioral Nutrition and physical Activity, Jan 15, 11:3.<br>- Sarphati Amsterdam (2016). Sarphati Cohort. Geraadpleegd op 30 juni 2016, van https://www.sarphati.amsterdam/#sarphati-cohort.

	- Schönbeck, Y., Talma, H., van Dommelen, P., Bakker, B., Buitendijk, S.E., Hirasing, R.A., & van Buuren, S. (2011). Increase in prevalence of overweight in Dutch children and adolescents: a comparison of nationwide growth studies in 1980, 1997 and 2009. PLoS One, 6(11), e27608.<br>- Schönbeck, Y., van Buuren, S. (2010). Factsheet Resultaten Vijfde Landelijke Groeistudie. Geraadpleegd op 25 mei 2016, van https://www.tno.nl/media/1996/20100608-factsheet-resultaten-vijfde-landelijke-groeistudie1.pdf<br>- Schulz, J., Henderson, S.E., Sugden, D.A., & Barnett, A.L. 2011. Structural validity of the movement ABC-2 test: factor structure comparison across three age groups. Research in Developmental Disabilities, 32(4), 1361-1369.<br>- Volksgezondheidenzorg.info. (2016). Gezondheidsgevolgen bij kinderen. Geraadpleegd op 25 mei 2016, van https://www.volksgezondheidenzorg.info/onderwerp/overgewicht/cijfers-context/gezondheidsgevolgen#!node-gezondheidsgevolgen-bij-kinderen
